# dRNA-Seq Reveals Genomewide TSSs and Noncoding RNAs of Plant Beneficial Rhizobacterium *Bacillus amyloliquefaciens* FZB42

**DOI:** 10.1371/journal.pone.0142002

**Published:** 2015-11-05

**Authors:** Ben Fan, Lei Li, Yanjie Chao, Konrad Förstner, Jörg Vogel, Rainer Borriss, Xiao-Qin Wu

**Affiliations:** 1 Co-Innovation Center for Sustainable Forestry in Southern China, College of Forestry, Nanjing Forestry University, 210037 Nanjing, China; 2 RNA Biology Group, Institute for Molecular Infection Biology, University of Würzburg, 97080 Würzburg, Germany; 3 Fachgebiet Phytomedizin, Albrecht Daniel Thaer Institut für Agrar- und Gartenbauwissenschaften, Lebenswissenschaftliche Fakultät, Humboldt Universität zu Berlin, 14195 Berlin, Germany; Max-Planck-Institute for Terrestrial Microbiology, GERMANY

## Abstract

*Bacillus amyloliquefaciens* subsp. *plantarum* FZB42 is a representative of Gram-positive plant-growth-promoting rhizobacteria (PGPR) that inhabit plant root environments. In order to better understand the molecular mechanisms of bacteria-plant symbiosis, we have systematically analyzed the primary transcriptome of strain FZB42 grown under rhizosphere-mimicking conditions using differential RNA sequencing (dRNA-seq). Our analysis revealed 4,877 transcription start sites for protein-coding genes, identified genes differentially expressed under different growth conditions, and corrected many previously mis-annotated genes. We also identified a large number of riboswitches and *cis-*encoded antisense RNAs, as well as *trans-*encoded small noncoding RNAs that may play important roles in the gene regulation of *Bacillus*. Overall, our analyses provided a landscape of *Bacillus* primary transcriptome and improved the knowledge of rhizobacteria-host interactions.

## Introduction

Rhizobacteria are a heterogeneous group of bacteria directly colonizing or living in the vicinity of plant roots. Many rhizobacteria are beneficial to plants as they stimulate plant growth and/or protect plants from phytopathogens. Rhizobacteria have been developed as microbial fertilizer and biocontrol agents. Understanding the symbiotic relationship between rhizobacteria and their host plants has been a longstanding research interest in environmental microbiology. Among a large variety of plant inhabitants, the Gram-positive rhizobacterium *B*. *amyloliquefaciens* subsp. *plantarum* FZB42 was extensively used as a model of plant-growth-promoting organisms to study the mechanisms of rhizobacterial biocontrol activity [1–6]. However, while genomic studies have revealed novel gene clusters and informed the pathways for the synthesis of natural products of this organism, our knowledge of the structure and expression of these genes has been restricted to a series of individual investigations [2, 4, 6–11].

In order to adapt to the fast-changing environment surrounding plant roots, rhizobacteria need a tuneable regulation system that can turn on or off gene expression in a dynamic fashion. To understand the mechanisms of such a gene regulation, it is critical to know where the transcription starts. Global mapping of transcription starting sites (TSSs) can facilitate the identification of promoter regions, *cis*-acting regulatory elements and cognate transcriptional regulators, and thereby helping to design reporter constructs. We here demonstrate the first global TSS map of a rhizobacterial *Bacillus* species using a recently developed differential RNA-seq (dRNA-seq) approach [12]. This approach can differentiate the 5’ end status between primary transcripts (5’-ppp) and processed transcripts (5’-p) by exploiting a terminator exonuclease (TEX) that only degrades 5' monophosphorylated RNA. By splitting a total RNA sample and treating each half with or without TEX, primary transcripts will be enriched in the TEX-treated samples due to the selective removal of processed fragments [12]. Then, the TSSs of primary transcripts are identified in a single-nucleotide resolution using next-generation sequencing technology. Similar approaches have been used to generate high-quality transcriptome maps of diverse bacterial species, including Gram-negative species such as *Helicobactor pylori* [12], *Escherichia coli* [13], *Salmonella typhimurium* [14], *Chlamydia pneumoniae* [15], *Xanthomonas campestris* [16], and Gram positive bacteria like *B*. *subtilis* [17] and *Mycobacteium tuberculosis* [18].

In addition to charting the landscape of expressed protein-coding genes, dRNA-seq also provides a tool for a rapid genome-wide discovery of small noncoding RNAs (sRNAs). Over the last decade, sRNAs have been shown to play important roles in bacterial adaptation to changing environments. To date, sRNAs have been extensively characterized in the Gram-negative model bacteria like *E*. *coli* or *Salmonella enterica* where their total count is approaching 300 [19–21]. Much fewer sRNAs have been functionally characterized in Gram-positive organisms. It has also been argued that riboregulation in Gram-positive bacteria relies on *cis-*acting elements such as riboswitches rather than *trans-*acting sRNAs, partially because the genetic inactivation of Hfq, an RNA chaperon facilitating baseparing interactions between sRNAs and their target mRNAs [22], seems to have a minor effect in Gram-positive species [23–26], as compared to Gram-negative bacteria. Nonetheless, increasing numbers of sRNAs have been identified in Gram-positive organisms [17, 27–40]. For example, 62 novel sRNA candidates were identified in the first RNA-seq study of *B*. *subtilis* [17], bringing the number of known sRNAs in this model organism to 80, with 30 of them having been confirmed experimentally [17, 31–40].

A few sRNAs in *B*. *subtilis* have been characterized in detail [29, 35, 41], and demonstrated as crucial regulators in diverse physiological circuits. For example, the SR1 sRNA (a.k.a. BsrF), which itself is activated by the *B*. *subtilis* transcriptional regulator CodY, represses the translation of the AhrC transcription activator thereby regulating arginine catabolism [35, 42]; the RnaC/S1022 sRNA modulates the expression of transcriptional regulator AbrB to suppress exponential growth and to promote heterogeneity of *Bacillus* population [41]. Given these established roles of sRNAs in modulating gene expression, we reasoned that sRNAs may also be active in bacilli that live in a complex environment such as the rhizosphere. Here, we present the primary transcriptome of *B*. *amyloliquefaciens* FZB42 grown under several different conditions. Our dRNA-seq analysis led to an identification of close to 5,000 TSSs in a genome with approx. 3,700 mRNA genes [1]. This improves our knowledge of gene regulation events that enable *Bacillus* to respond to environmental cues. Moreover, we identified and further experimentally validated 21 new sRNAs, whose functions remain elusive. To our knowledge, this is so far the most extensive transcriptomic study of plant associated *B*. *amyloliquefaciens*, a group of bacteria with fundamental importance in plant-microbe interaction.

## Materials and Methods

### Root exudates and bacterial cultures

**Fig 1:**
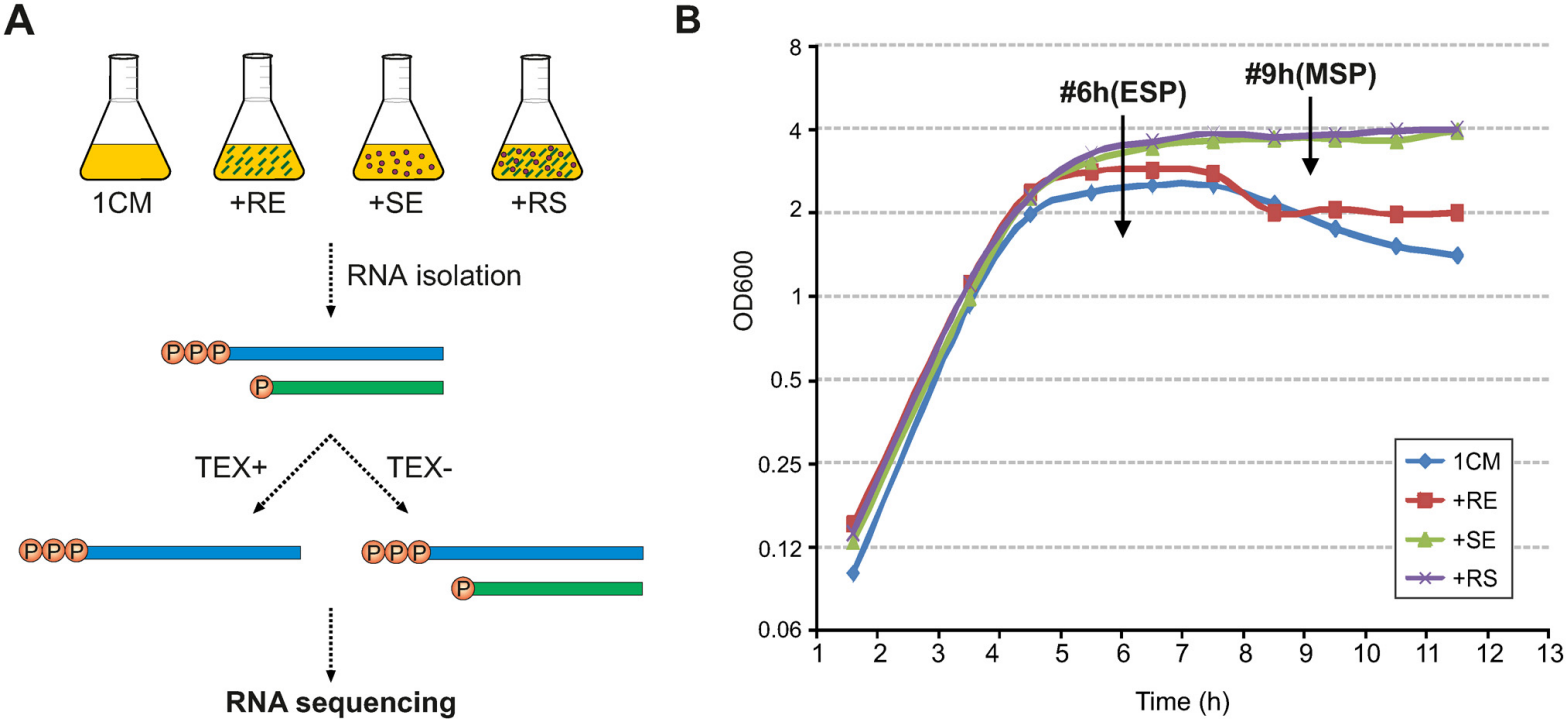
Growth conditions of *Bacillus amyloliquefaciens* FZB42. (A) Schematic diagram of the dRNA-seq workflow of *B*. *amyloliquefaciens* FZB42. FZB42 was cultured in four media: i) 1CM medium; ii) 1CM medium supplemented with maize root exudates (+RE); iii) 1CM medium supplemented with soil extract (+SE); iv) 1CM medium supplemented with both maize root exudates and soil (+RS). (B) Growth curves of FZB42 growing in the four media. The cultures were collected for total RNA preparation at different time points (#6h & #9h) as indicated by the arrows. TEX: terminator exonuclease; ESP: early stationary phase; MSP: middle stationary phase.

*B*. *amyloliquefaciens* FZB42 was cultured under four conditions [43]: i) in 1CM medium (1% peptone, 0.05% yeast extract, 0.5% NaCl); ii) 1CM medium supplemented with maize root exudates (RE) of 0.25 mg/ml; iii) 1CM medium supplemented with 10% soil extract (SE) prepared from soil collected from the farmland (with the permission of farmland owner Mrs Constanze Ackermann) in Wuerzburg, Germany; iv) 1CM medium supplemented with both the maize root exudates (0.25 mg/ml) and the soil extract (10%). The maize root exudates were collected from the cultivar “DengHai 11” as described in our previous work [43]. The cultures were incubated at 210 rpm and 28°C and collected at early stationary phase and at middle stationary phase, respectively, for total RNA preparation (Fig 1).

### Total RNA preparation

The bacterial cells harvested were immediately mixed with 0.2V STOP buffer (95% ethanol + 5% phenol). After a quick shake, the cultures were snap-frozen in liquid nitrogen and then stored at -80°C until RNA isolation. To isolate total RNA, the frozen cultures were half-melted on ice and then centrifuged at 5,000rpm for 10 minutes at 4°C. The pellets were suspended with ice-cold Trizol^®^ agent and then transferred to a cryo-tube filled with 0.2V sterile glass beads of 0.1 mm in diameter. Subsequently, the mixtures were milled on FastPrep^®^-24 system for twice of 30 seconds at speed of 6.0 m/s with a one-minute interval cooling on ice. The milled cells-containing Trizol^®^ agent was continued to isolate total RNA as previously described [44].

### Deep sequencing

The isolated RNA samples were initially quality filtered, and then used to construct cDNA libraries by Vertis Biotechnologie AG (http://www.vertisbiotech.com), Germany, as described in the supporting information of reference [12]. Sequencing was performed on a Solexa HiSeq 2000 machine. Raw cDNA reads were quality trimmed and poly-A clipped. The remaining clean reads were mapped to the *B*. *amyloliquefaciens* FZB42 genome (NC_009725.1) using READemption (version 0.3.4) with “create”, “align” and “coverage” subcommands. The align subcommand was called with the parameters (—split -r -p 24 -a 95 -l 12—poly_a_clipping –progress) [45]. The coverage per nucleotide was calculated, normalized by the number of total number of mapped of reads for each library and visualized in the Integrated Genome Browser [46].

### TSS prediction and differential expression analysis

TSSs were predicted by TSSpredator [47] which use a ratio-based approach to identify the enriched sites in TEX treated samples compared to TEX untreated samples. Based on their locations relative to annotated genes, TSSs were classified as primary TSS, secondary TSS, antisense TSS, internal TSS and orphan TSS. The TSS with the strongest expression and within 500 nucleotides upstream of a gene was considered as primary. The TSS that associated with same gene was considered as secondary. Antisense TSS was located internal or within 100 nucleotides of a gene on the antisense strand, while internal TSS was located inside a gene on the sense strand. The other TSS without any annotated gene nearby was orphan TSS. To identify the differential expressed genes under two different conditions (A&B), we defined the differential expressed genes as those passing three filters: i) fold change of Reads Per Kilobase Per Million mapped reads (RPKM) under two conditions (condition A and condition B) was greater than 2 (for simplicity, we refer to this as RPKM_A/B > 2 throughout the article), and ii) RPKM of the gene in condition A was greater than 2 (refer to RPKM_A > 2), and iii) raw reads of the gene in condition A was greater than 50 (refer to as Raw reads_A > 50). The RPKM values were calculated from the untreated librarys.

### Radioactive labelling of oligonucleotides

Oligonucleotides were radioactively-labelled at their 5'-OH ends by T4 polynucleotide kinase (T4 PNK) that catalyses transfer of γ-phosphate from ^32^P-ATP. For this, 40 pmol of oligos were mixed with 4 μl [γ-^32^P] ATP (10 μCi/ml) and phosphorylation took place by incubation of the mixture with T4-Kinase at 37°C for 30 minutes. The reaction was stopped by heat inactivation at 70°C for 10 minutes.

### sRNA detection by northern blotting

The total RNA samples were separated (10 μg/each sample) on 6% PAA 7M urea gel in 1×TBE buffer. The samples were denatured at 95°C for 5 minutes and then cooled on ice for another 5 minutes. After running the RNAs were transferred to a positively charged nylon membrane. Finally the RNAs were immobilized on the membrane by cross-linking using UV radiation. The membrane was initially incubated in 15 ml QIAquick hybridization buffer for 1 hour at 42°C, while the radioactively-labelled oligo probes were denatured at 95°C for 5 minutes and then immediately cooled on ice to unfold the secondary structures. Subsequently, the membrane was hybridized overnight with 1 μl denatured oligo probes at 42°C. The membrane was washed three times at 42°C, each for 15 minutes, with 2× SSC/0.1% SDS, 1× SSC/0.1% SDS, and 0.5× SSC/0.1% SDS, respectively. The results were visualized by Typhoon FLA 9500 scanner (GE Healthcare).

### KEGG Pathway Analysis

The differentially expressed genes with various fold changes (logarithmic) were mapped into KEGG pathway using the GSEA method [48], a gene set enrichment analysis based approach for determining the significant pathways. The significant pathways with *p*-value smaller than 0.1 were displayed using Pathview [49].

### Data deposition

Raw sequence reads were stored in NCBI Gene Expression Omnibus (GEO) with accession number GSE66681.

## Results

### Genome-wide TSS Analysis

In order to mimic natural plant-root environments and to promote the expression of genes important in rhizosphere ecosystem, we cultivated *B*. *amyloliquefaciens* FZB42 in 1CM medium supplemented with either maize root exudates (RE), or soil extract (SE), or both (RE+SE, a.k.a. “RS”). We determined the kinetics of bacterial growth under four different conditions and collected bacterial cultures for total RNA isolation at the indicated time points (Fig 1). Based on the growth curves, the sampling points at ~6 hours roughly correspond to the early stationary phase (ESP) for SE and RS samples, and to the middle stationary phase (MSP) for 1CM and RE samples. Since we wanted to identify genes affected by RE at a similar growth stage, two additional samples were further collected at MSP under SE and RS conditions (at ~9 hours), resulting in six bacterial samples in total. For simplicity, we hereafter refer to these samples using medium features (*i*.*e*., 1CM, RE, SE, RS) and sampling time (*i*.*e*., 6h or 9h) connected with a “#” symbol, *e*.*g*., 1CM#6h (S1 Table). Following the published dRNA-seq cDNA synthesis protocol [12], we split each total RNA sample into two aliquots, one of which was treated with terminator exonuclease (TEX). A total of 12 cDNA libraries were constructed and deep sequenced to ~120 million reads using Illumina technology.

**Fig 2:**
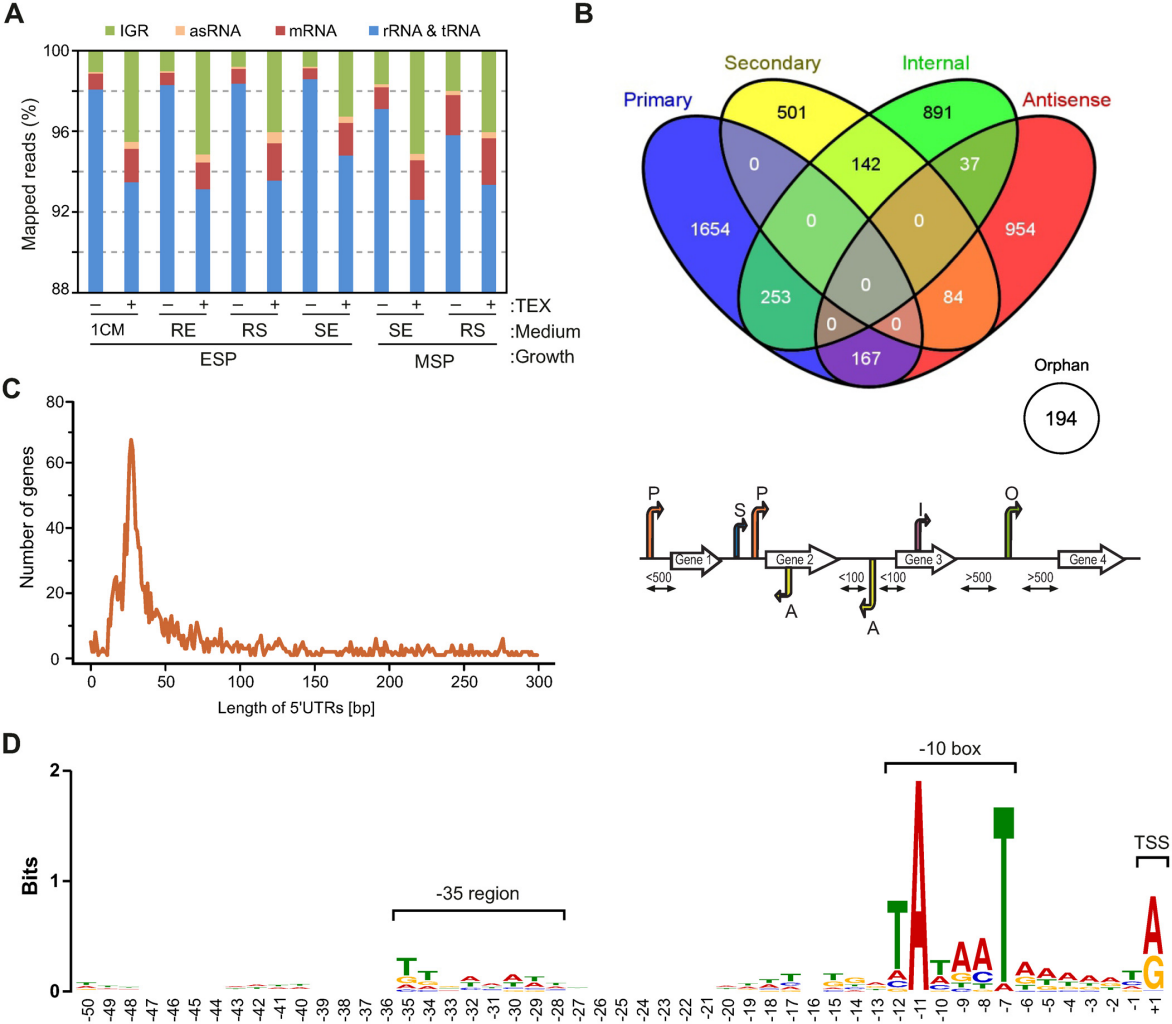
General profile of the sequencing data. Distribution of the mapped reads of various RNAs from each library varying in different culture conditions or terminator exonuclease treatment. 1CM: 1CM medium; +RE: 1CM medium supplemented with maize root exudates (RE); +SE: 1CM medium supplemented with soil extract (RE); +RS: 1CM medium supplemented with both maize root exudates and soil (RS); TEX: terminator exonuclease; ESP: early stationary phase; MSP: middle stationary phase. (B) Venn diagram showing overlaps among various TSS categories. The TSS were designated into the categories as defined in [12]. (C) Length distribution of 5’leader sequence in *B*. *amyloliquefaciens* FZB42. (D) Motif analysis among upstream of TSSs of *B*. *amyloliquefaciens* FZB42. Sequences upstream of TSSs were extracted (positions -1 to -50) and common sequence motifs were searched using MEME software. The coordinates give positions relative to TSSs.

After a standard quality check and adaptor removal, cDNA reads were mapped to the FZB42 genome using the READemption pipeline [45]. The number of reads allocated to each gene annotation was quantified and the sum of reads of each RNA class was calculated (S1 Table). We have observed a high mappability for all these cDNA libraries as most reads (>91%) could be mapped to the FZB42 genome. As expected, the TEX-treated (TEX+) libraries contain a lower percentage of housekeeping rRNA/tRNA reads (Fig 2A), arguing that the TEX treatment successfully depleted processed transcripts (mostly rRNA/tRNA). We have detected 4,877 TSSs in total, which were grouped into five different categories according to [12], including 2,074 (43%) primary TSSs, 727 (15%) secondary TSSs, 1,323 (27%) internal TSSs, 1,424 (25%) antisense TSSs, and 194 (4%) orphan TSSs (S2 Table). As expected, some TSSs belong to multiple categories (Fig 2B).

The 2,074 primary TSSs detected in all six conditions were assigned to transcripts for ~56% of the annotated 3,701 genes in the FZB42 genome. We randomly selected 10 genes with experimentally mapped TSSs to verify the reliability of TSS designation. The result showed that our analysis accurately captured all these known TSS, confirming the robustness of our TSS annotation (S3 Table). Eighty of the primary TSSs were detected in only one specific condition, predominantly (71, ~90%) in the SE#9h condition (S4 Table). The assigned primary TSSs allowed us to determine the 5’ untranslated region (UTR) for 1,958 mRNAs. The majority of mRNAs possess a 5’UTR sequence between 10 to 50 nucleotides in length (Fig 2C), indicating an optimal arrangement to support efficient translation in *Bacilli*, similar to the situation in many Gram-negative bacteria [12, 15, 50]. 445 mRNAs carry a longer 5’UTR sequence (>100 nucleotides) which may harbor potential regulatory elements for post-transcriptional regulation *in cis* or *in trans*. This number should be taken with some caution, though, since many of these genes (>50%) encode hypothetical proteins whose start codon annotation may need correction. By contrast, 28 mRNAs possess no or a very short 5’UTR (<10 nucleotides) and should be considered leaderless mRNAs (S5 Table).

Sequence analysis of the nucleotide composition for primary TSS revealed a strong selection of purines (A or G) at the +1 site, which is required for efficient transcription initiation by RNA polymerase, at least in many Gram-negative bacteria [51, 52]. Using all identified TSSs, we aligned their upstream sequences to identify potential elements signaling transcription activity. A typical TATA box (TATAAT) was identified at the -10 region, which further supported the high accuracy of our TSS mapping. A weaker motif (TTnAAAtt) is observed at the -35 region, which is slightly different from the canonical TTGACA sequence in *B*. *subtilis* (Fig 2D). This may be due to a large assembly of different promoter sequences that are recognized by a variety of sigma factors.

Secondary TSSs are operationally defined as a TSS being located downstream of primary TSS but having fewer cDNAs [12]. Gene expression initiating from secondary TSSs indicates alternative promoters. More than 30% of the total secondary TSSs (727) were detected in each condition, while a higher percentage of 65% and 80% were found in conditions SE#9h and RS#9h, respectively (S6 Table). Excluding the overlap between different conditions, *i*.*e*., only considering those genes specifically expressed in one condition, we identified a total of 95 secondary TSSs, which mostly fall in the conditions of SE#9h and RS#9h (28 and 59 TSSs, respectively). The major portion of secondary TSSs unique to condition SE#9h and RS#9h precedes a gene encoding a hypothetical protein (S6 Table), similar to our observation that two thirds of the primary TSSs detected only in SE#9h precede a gene of unknown function. Such information implies that a large part of the expressed genes at middle stationary phase remain elusive with respect to function.

### Differentially expressed genes at two growth phases

Upon entering stationary phase, *Bacillus* species initiate the sporulation gene expression programme to prepare for production of spores. The relevant RNA samples SE#9h and RS#9h were isolated from MSP while samples SE#6h and RS#6h were isolated from ESP. Therefore a comparison ofgene transcription between the two time points will allow us to identify sporulation-associated genes differentially expressed in the two different growth stages. For scoring differential gene expression, we focused on transcripts passing our strict abundance filters (see experimental procedures). We identified 208 and 433 genes for the SE and RS conditions, respectively, showing strong up-regulation at MSP compared to ESP. 148 activated genes were common to both the SE and RS conditions. Nearly 40% of the common genes are of unknown function while 27% of them are associated with sporulation (S1 Fig).

Down-regulated genes at MSP were rare (5 genes in SE condition and 23 genes in RS condition) and only three (*spoIIB*, *spoIIE*, and *bpr*) of these overlapped in both conditions. The down-regulation of the three genes is consistent with previous studies [53, 54] where they were reported to drop in expression after sporulation start, roughly corresponded to MSP here.

To further characterize the 151 common genes (148 up-regulated, 3 down-regulated; S7 Table) that may be relevant to the sporulation process, we examined their upstream sequences for conserved binding sites of RNA polymerase sigma factors. Using knowledge from *B*. *subtilis*, we determined sigma factor binding motifs in 37 genes, 33 of which are known to be regulated by SigK or SigG. Moreover, based on the TSSs identified in this study, we predicted with the online tool [55] binding motifs for another 36 genes, 24 of which are also regulated by SigK or SigG (S7 Table). Activation of SigG in the forespore precedes and is required for the subsequent activation of SigK in the mother cells. After activation, SigG and SigK are responsible for transcription of a series of genes in the forespores and the mother cells, respectively [56, 57]. No binding motif is predicted for the remaining half of the common genes, partly due to lack of defined TSSs. The high percentage of SigG/SigK-dependent genes (57 out of 73) suggests that the time of #9h sample roughly corresponded to stage III-IV (completion of engulfment and cortex synthesis) of the seven stages of sporulation cycle [58, 59]. This deduction in turn supports the suitability of our above designation the time of #9h as MSP.

### Riboswitches and *Cis-*encoded antisense RNAs

*Cis*-acting regulatory RNA elements encoded within 5' UTRs are prevalent in Gram-positive bacteria [60, 61]. By quering all 5'-UTRs longer than 70 nt against the Rfam database [62, 63], we identified 53 cis-encoded RNA elements (S8 Table) which include various conserved riboswitches (TPP, SAM riboswitch, purine riboswitch, glycine riboswitch, FMN riboswitch, preQ1 riboswitch, glmS riboswitch) and other leader sequence such as L10-, L13-, L19-, L20, and L21-leader, T-box leader, *Trp* leader_2, *ykoK* leader, *ykkC-yxkD* leader [64], pan RNA motif [65], yjdF RNA [65], and PyrR binding site [66]. The 53 RNA elements validated most of the *cis*-regulatory riboswitches or leader sequences provided by the Rfam database for *B*. *amyloliquefaciens*, except *ylbH* leader and *pyrG* leader. The absence of these RNAs is probably due to a low expression of them in our specific growth conditions.

Antisense RNAs (asRNAs) regulate bacterial gene expression *in cis*, often by forming long RNA duplexes with the oppositely encoded mRNAs [67]. Manual inspection identified a total of 136 antisense RNA candidates, 90 of which are located antisense to coding regions (S9 Table). The remaining 46 antisense RNA candidates are opposite to a 5’UTR or 3’UTR (S10 Table). Eight asRNA candidates showed higher expression in the #9h samples as compared to the #6h samples (S11 Table); only two of them are opposite to genes of known function (*yocH* and *sspF*), both of which are specifically expressed at stationary growth phase.

### Gene re-annotation and operon prediction

A group of 46 highly expressed intergenic transcripts were detected. We identified six of them as tRNAs according to annotation in Rfam, including four that were previously annotated to encode hypothetical proteins. We blasted the remaining 40 ranscripts against the non-redundant protein database for homologous proteins that have been annotated in other bacterial genomes. The blast results suggest that the 40 transcripts encode small proteins (30–99 aa), only a few of which have been functionally characterized. For example, we have detected *pznA*, predicted to encode a 41-aa protein and recently shown to be involved in synthesis of an antimicrobial peptide [3, 4]. Further, we found eight TSSs within 50 bp downstream of previously annotated start codons. Sequence analysis revealed a new start codon locateing at 40–100 bp downstream of their TSSs and a plausible ribosomal binding site (RBS) locating at ~7 bp upstream of the new start codons. Accordingly, we propose to change the annotation for these eight genes, and for two other transcripts bearing an ORF antisense to previously annotated genes (S12 Table). Overall, we propose to add or re-annotate 46 undefined genes to the genome annotation of FZB42 (S13 Table) and to correct 10 gene annotation. In addition, we failed to detect any reliable reads in the coding region of gene *yhaY* (222 bp) may not exist since we failed to detect any reliable reads in its coding region but found a highly expressed transcript on its antisense strand. The expressed transcript is 223 nt in length and contains no ORF, suggesting it could be an sRNA which may silence *yhaY* completely in our tested conditions.

Some of these short transcripts from intergenic regions (IGRs) may have dual functions, acting as both mRNA and regulatory RNA. For example, two of the small protein coding transcripts (*rho-glpX*, *yrzI-yrhG*) were previously identified as sRNAs [17]. Another two short IGR transcripts (*xylB-pps*, *cypC-yitS*; see S13 Table) containing an ORF are located antisense to the 3’UTR or terminator regions of their neighbor genes, indicating they may also function as an sRNA regulating *in cis* the expression of the neighboring genes.

**Fig 3:**
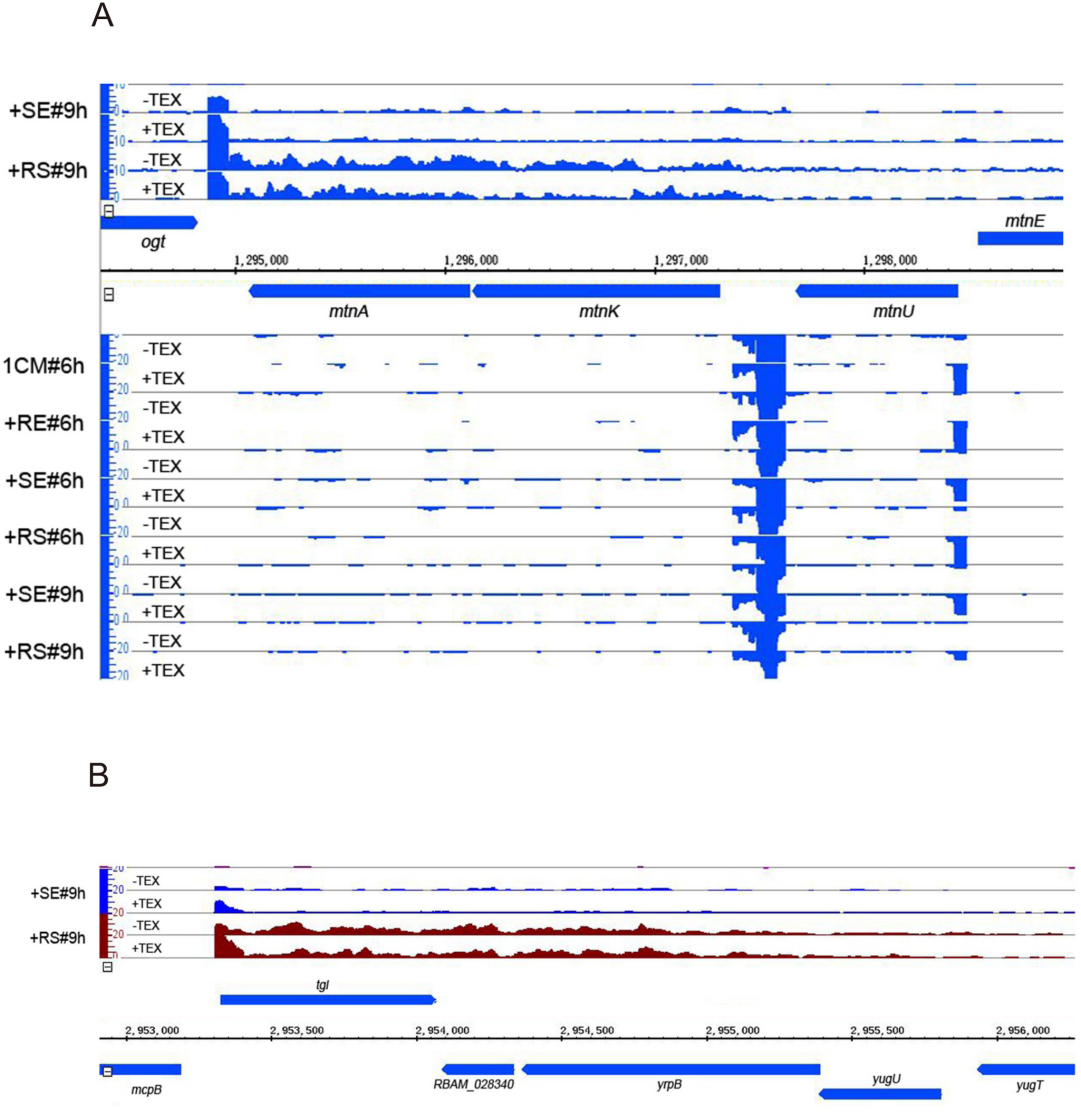
A subset of operons antisense to known genes. Two operons identified antisense to a known operon (A) or to known genes (B). cDNA reads in each library without (-TEX) or with (+TEX) terminator exonuclease treatment were mapped to *B*. *amyloliquefaciens* FZB42 chromosome and visualized by IGB 8.1. Vertical bars indicate cDNA recovery detected in each library and were adjusted to the same scale of 20. The genes indicated above the horizontal coordinates are located on the forward strand while the genes indicated below the horizontal coordinates are located on the reverse strand. Bacterial cultures were sampled for total RNA extraction at two time points (#6h&#9h) from four conditions: i) in 1CM medium; ii) 1CM medium supplemented with the maize root exudates (+RE); iii) 1CM medium supplemented with soil extract (+SE); iv) 1CM medium supplemented with both maize root exudates and soil extract (+RS). The time point #6h corresponded to early stationary phase while #9h corresponded to middle stationary phase.

We found 730 operons for the FZB42 genome predicted by the DOOR database [68]. We manually checked the 730operon loci with our visualized dRNA-seq data and thus validated 78 polycistronic transcripts covering a total of 210 genes (S14 Table). Most of the confirmed operons (53 out of the 78) are bicistronic. Intriguingly, we observed two long asRNA that resemble excludon structures (Fig 3A and 3B). Excludon is a transcriptome organization where divergent genes with mutually exclusive or related functions transcribe long antisense RNAs in order to suppress the expression of the other gene in the same locus [69, 70]. In addition, we found that 13 genes, which are listed within a polycistronic operon by DBTBS [55], were actually transcribed as a monocistronic unit (S15 Table). This indicates that alternative TSSs could be used to initiate their gene transcription in a certain environment. For example, the polycistronic operon of *yhaA-yhfA-yhgC* varied their transcript initiation from different TSSs at ESP (#6h) or MSP (#9h) (S2 Fig).

### Identification of *trans-*encoded small RNAs

sRNAs are known to be encoded by independent genes located within IGRs. To identify such sRNAs in FZB42, we screened over 500 abundant IGR transcripts (with more than 50 raw reads), excluding those extending from or into coding regions of their flanking genes. After further filtering out mis-annotated mRNAs, riboswitches and other leader sequences, or antisense RNAs, as explained above, we determined 86 candidate sRNAs. Thirteen of them were known sRNAs, which include the FZB42 homologes of three ubiquitous RNAs (M1 RNA of RNase P, 6S RNA [there is only one gene copy in strain FZB42], and tmRNA) and of seven experimentally confirmed sRNAs in *B*. *subtilis* (FsrA, RsaE, BsrG, BsrF, BsrI, polC-ylxS, and ncr1175). Three more potential homologs of *B*. *subtilis* sRNAs were detected in the IGRs of *ymzA-nrdI*, *yvcI-trxB*, and *yneK-cotM* [17]. By contrast, we failed to detect potential homologs of the the BsrC or SurA sRNAs [31, 38]. BsrC was expressed in *B*. *subtilis* during the vegetative phase but undetectable during the stationary phase [38], which may explain why it was missing in our analysis; SurA, a ~280 nt-long sRNA from the IGR between *yndK* and *yndL* in *B*. *subtilis* [31], is not conserved in FZB42 probably due to genomic re-arrangement. Though FZB42 encodes a *yndL* homolog, the other flanking gene *yndK* is absent. However, a part of the SurA locus is expressed as a 94 nt-long transcript antisense to the *yndL* mRNA (S3 Fig).

**Fig 4:**
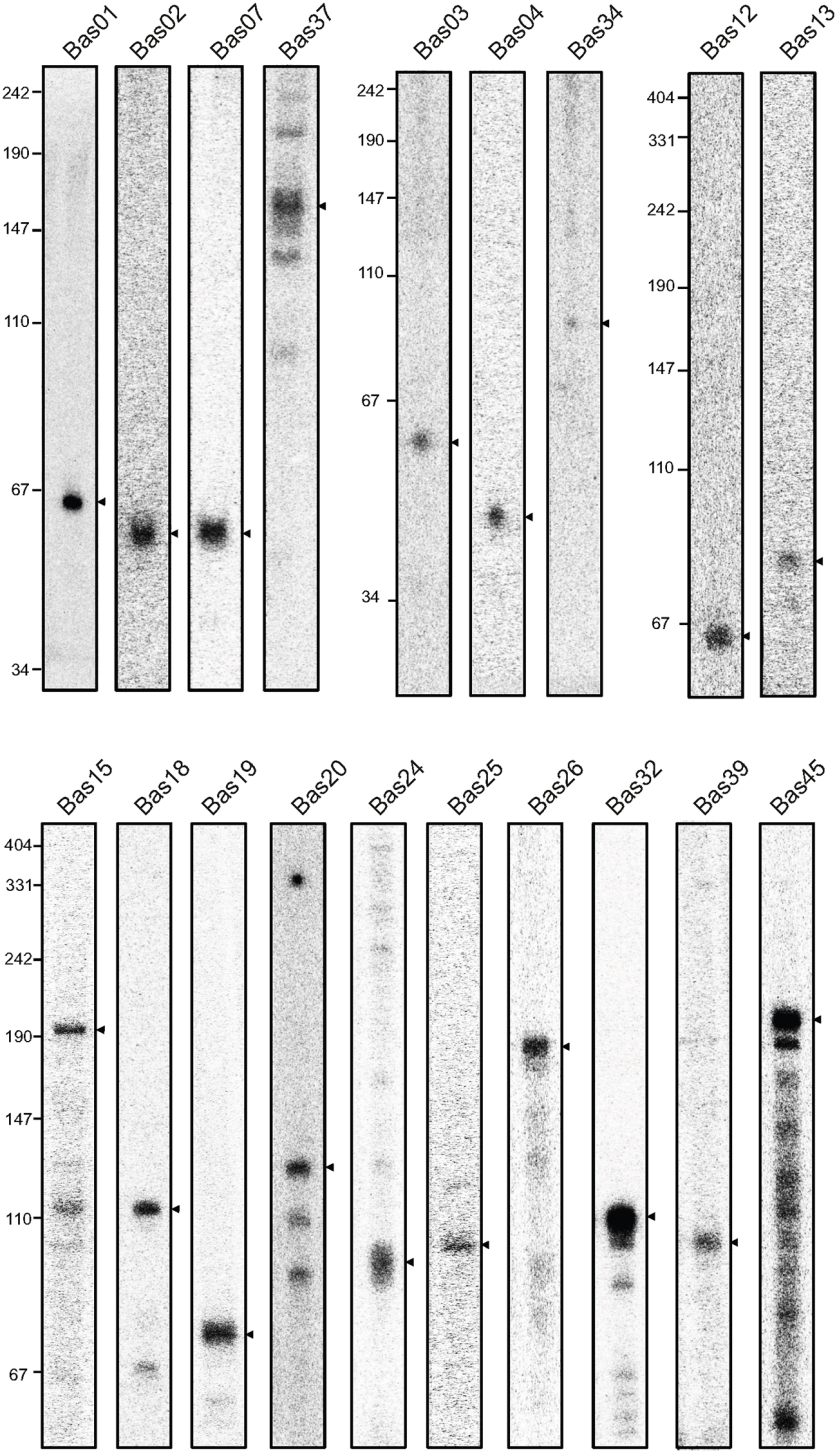
Verification of sRNAs in *B*. *amyloliquefaciens* FZB42. sRNAs identified by sequencing data were validated by Northern blot. One representative lane was displayed for each sRNA identified. The names of sRNAs and the molecular marker were indicated on the top and on the left respectively. For more details of each sRNA see S6 Fig.

To validate our results, we probed on Northern blots 35 selected sRNA candidates, 19 of which were detected as distinct transcripts (Fig 4 and S16 Table). One of the candidates (Bas28) was also detected but with additional shorter bands, indicating sRNA processing (S4 Fig). A distinct transcript of >500 nt in length was identified in the *trpS-oppA* IGR and designated as Bas35; in this case, the detected transcript much exceeded the size predicted by the RNA-seq data (~95nt) (S5 Fig). Since we failed to find a convincing ORF on the sense strand within the IGR, Bas35 is probably a long noncoding RNA covering5’-UTR of *oppA*. Intriguingly, Bas35 is located antisense to the T-box leader upstream of *trpS*, suggesting it may possess a regulatory role *in cis*.

The expression profiles of the new sRNAs along growth in different media give valuable hints of how these sRNAs are regulated by rhizosphere enviroments. Four sRNAs (Bas02, Bas03, Bas24, and Bas39) seemed to be affected by root exudates, and almost a half of all sRNAs (Bas02, Bas04, Bas19, Bas20, Bas23, Bas24, Bas26, Bas32, Bas39) were either positively or negatively regulated by soil extract (S6 Fig). Furthermore, many sRNAs (Bas01, Bas02, Bas07, Bas12, Bas19, Bas20, Bas26, Bas32 and Bas45) strongly accumulated in MSP (S6 Fig), with several of them showing the highest expression peak at the last sampling time point. These sRNAs may be involved in stationary phase-specific processes such as sporulation. For Bas26 (*yjcA-RBAM_01181*0) (S7 Fig), the intergenic region where its major cDNA reads occurred belongs to 3’UTR region of *yjcA* [71]. Thus we deduced that Bas26 could be a 3’UTR-derived sRNA, a demonstrated phenomenon in Gram-negative bacteria that 3’UTR serve as a genomic reservoir of sRNAs [44].

### Expression of genes involved in bacterium-plant interaction

*B*. *amyloliquefaciens* FZB42 is a model strain of plant beneficial rhizobacteria. To find out how FZB42 responds to root environment, we compared the gene expression profile from the RS and SE conditions at MSP (#9h). Our analysis revealed 361 genes that were regulated by the presence of maize root exudates. These genes, belonging to various functional categories, were mostly up-regulated by root exudates. For example, two of these genes, *tatCY* and *secY*, encoding components of Tat pathway and Sec pathway respectively, have been promoted in transcription. Their up-regulation indicates that FZB42 increased its secretion activity in the presence of root exudates. Only two genes were down-regulated by the root exudate; one encodes an endospore development protein (*spoIIB*) and the other one (*ywcI*) is of unknown function.

Among the 361 genes, 63 are reportedly implicated in plant-bacterium interactions (S17 Table). Our results supported this implication and, further, provided more information like TSSs, favoring future investigations on them. For example, FZB42 impressively devotes >8.5% of its genome to synthesis of antimicrobial metabolites [1, 4, 6], an important mechanism that many rhizobacteria protect plants from phytopathogens. The operons for three antimicrobial metabolites (bacillomycin D, plantazolicin, and difficidin) showed higher transcription (RPKM>2) than the others. We confirmed the published TSS of *bmyD* [7], but identified the previously unknown TSSs for plantazolicin and difficidin gene clusters (S8 Fig). Further, we identified the TSS of *yczE*. This highly transcribed gene encodes a putative membrane-spanning protein that is essential for the synthesis of difficidin, bacillaene, macrolactin, and bacillomycin D [7] (S8 Fig). In addition, our data showed that bacillomycin D and plantazolicin were expressed mainly in the #6h but not in the #9h samples, while difficidin was expressed at both the #6h and #9h time points.

Other highly expressed genes in S17 Table include those involved in degrading plant derived organic substrates, such as celluloses, semicelluloses, chitin, opines, and plant proteins; the induction of these genes reflects the ability of rhizobacteria to utilize plant-derived nutrients which are essential for them to thrive in the rhizosphere. Some genes involved in swarming motility and biofilm formation were also highly expressed (S17 Table). These genes are usually related efficient colonization of plant roots, a prerequisite for rhizobacteria to interplay with plants. A FZB42 unique gene cluster (*RBAM_007740* to *RBAM_007770*) with no counterpart in *B*. *subtilis*, encodes proteins containing a collagen-related GXT structure motif. This cluster, required for biofilm formation and adhesion to plant roots [72], was highly expressed in #9h but not in #6h samples (S8 Fig). Further, some genes involved in response to oxidative stress (e.g., *sodA* and *tpx*) or adaption to atypical stresses (*cspB*, *cspC*, and *cspD*) were also highly induced; these genes may help the rhizobacteria to survive the plant basal defense.

## Discussion

**Fig 5:**
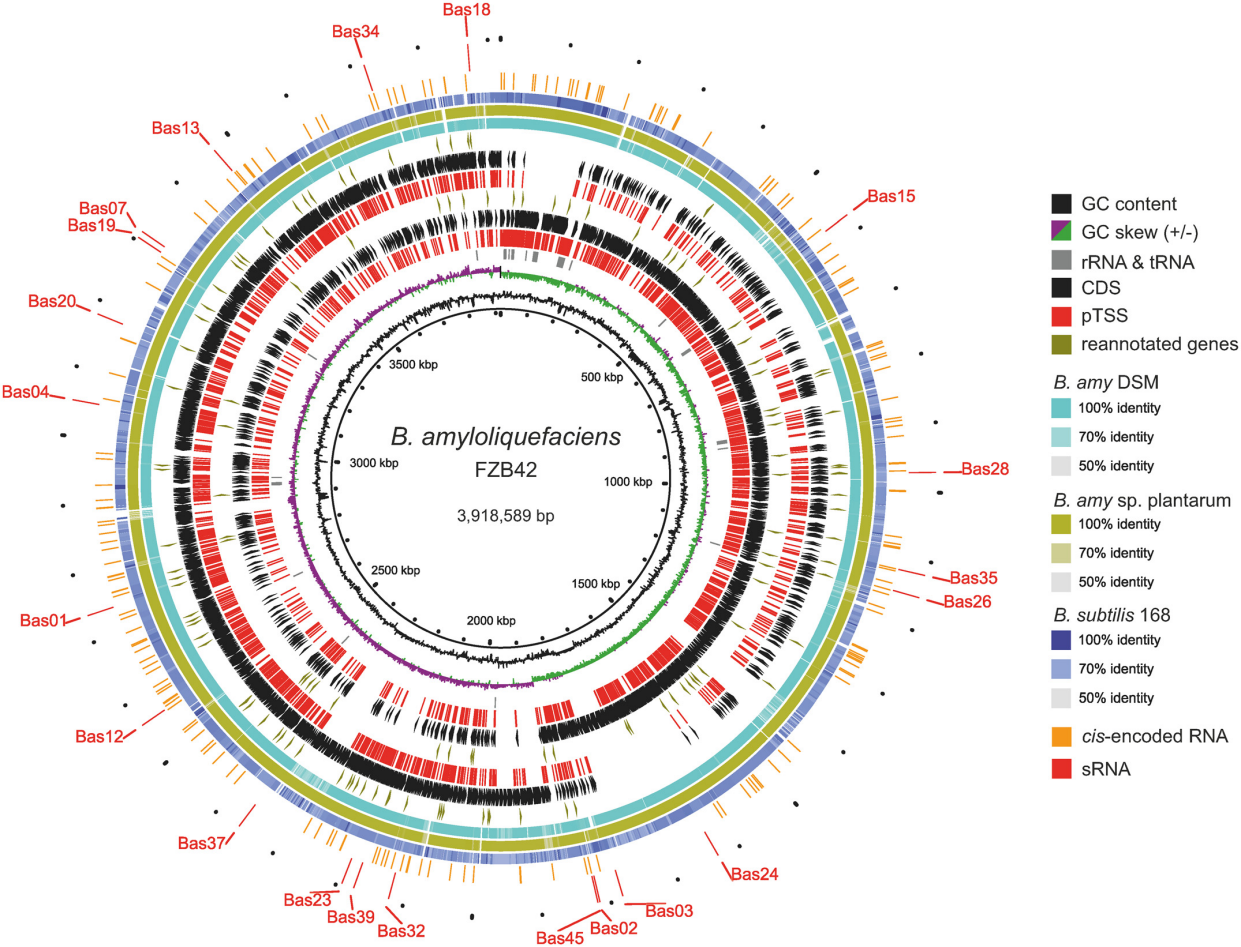
Genomic Visualization of various RNAs identified. The 1st ring from inside depicts genomic size maker; 2nd ring in black indicates DNA GC content; 3rd ring in purple and green indicates GC skew (+/-); 4th ring in grey indicates housekeeping rRNA and tRNAs; 5th and 6th ring indicates primary TSS (in red) and genes located on the leading strand (black arrows clockwise), 7th ring with oliver arrows indicates the re-annotated genes on the leading strand, including corrected CDS, leaderless mRNAs and proposed operons; 8th and 9th ring indicates primary TSS(in red) and genes (black arrows counterclockwise) located on the lagging strand; 10th ring with Olive green arrows indicates re-annotated genes on the lagging strand; 11th-12th ring show nucleotide conservation patterns with genomes of *B*. *amyloliquefaciens* DSM7 (NC_014551.1), *B*. *amyloliquefaciens* subsp. plantarum CAU B946 (NC_016784.1) and B. subtilis 168 (NC_000964.3), respectively; 13th ring in orange depicts all the cis-encoded RNAs annotated in this study, including riboswitches and all the antisense RNAs; 14th ring (the most outer-ring in red) depicts experimentally validated sRNAs in this study and their names are labeled.

*B*. *amyloliquefaciens* FZB42 is a Gram-positive representative of rhizobacteria that colonize plant roots and promote plant growth. Understanding its transcriptome structure and dynamics is critical to study the symbiotic relationship between rhizobacteria and host plants. In this work we have employed dRNA-seq and investigated the primary transcriptome of strain FZB42 under several conditions mimicking plant root environments. Our analyses established a comprehensive transcriptome structure for FZB42 (Fig 5) by determining primary TSSs for 60% of all FZB42 genes, assigning 5'-UTRs and operon structures, and identifying a large number of *cis*-encoded and *trans*-encoded RNAs which may be regulators of gene expression in *B*. *amyloliquefaciens*. Our analyses at the transcriptome level also improved the genome annotation of FZB42; for example, we proposed 46 new genes and corrected 10 mis-annotated genes (S13 Table).

In contrast with genome annotation, the structure of most bacterial transcriptomes has not been well-defined, mainly because of the lack of high-throughput approaches. Traditionally, bacterial TSSs were determined for a handful of well-studied genes using low-throughput methods such as primer extension or 5’-RACE. These methods are generally time-consuming and come with low resolution due to the gel-based system. The dRNA-seq based genome-wide TSS determination applied here generated a high-resolution transcriptome structure and revealed a complexity of gene organizations in bacterial transcriptome. Many coding genes are transcribed from multiple TSSs and may possess several different UTRs; and many mRNAs have overlapping and antisense transcripts (S3, S4 and S8–S10 Figs). For instance, the *yqzM* RNA encoding a 46-AA conserved hypothetical protein (S13 Table) was found complementary to the 5’UTR of *holA* as well as the 3’UTR of *comEC* (S9 Fig). We suspect that *yqzM* RNA and *holA/comEC* might also serve as antisense RNA regulating the expression of each other. Similarly, a large part of the *ytkA* mRNA (>200 bp) is antisense to another peptide-encoding transcript on the opposite strand (S10 Fig), suggesting another case of potential antisense regulation *in cis*.

Another characteristic of the bacterial transcriptome structure is its high plasticity under different conditions. We note that our current analyses are restricted to stationary phase, which is of high interest because most stress responses and noncoding regulators are activated under this condition. The transcriptome profile is very likely to differ from that in exponential growth, as exemplified by the dynamic expression profile of several sRNAs throughout bacterial growth (S6 Fig). As the sequencing cost continue to drop, full transcriptome analyses of bacterial samples from different growth stages and arrays of stress conditions will become feasible in the future.

In this study, the cultures were sampled from different growth stages and FZB42 was grown in different media supplemented with soil extract and/or maize root exudates in order to mimic rhizosphere environments. While soil extract is important for the growth of soil bacterial [73], root exudates are composed of a wide range of organic compounds and known to be the crucial players mediating the interaction between plants and rhizobacteria [74, 75]. These different conditions allow us not only to elucidate the complexity of FZB42 transcriptome structure as stated above but also to compare their gene expression in response to diverse influential factors. For example, we identifed a number of differentially expressed transcripts with respect to growth phase or root exudate. Since our sequence output contains many rRNA/tRNA reads (rRNA depletion would be beneficial for future sequencing analyses to obtain higher reads coverage), we applied a strict filter (see material and methods part) to select genes with high number of reads for reliable quantification and comparasion of gene expression, though this would compromise the detection of genes with low expression levels. The differentially expressed genes we identified form a useful resource meriting our attention for future work, in order to unravel molecular details of growth phase specific physiology or plant-bacteria communication.

Further, relating the expression of these genes with specific environmental or physiologic conditions would facilitate exploration of their function, regulation and relevance. For example, a number of genes up-regulated were enriched in the pathways of histidine (S11 Fig) or arginine metabolism (S11 Fig). In these two pathways, many genes seem upregulated at #9h than at #6h, which suggests enhanced synthesis of L-glutamate and arginine at MSP. This is in accordance with a previous report that L-glutamate and L-arginine are two predominant amino acids in spores of *B*. *subtilis* [76]. Together, these observations suggest a critical role of glutamate in spore formation, dormancy, or germination. The regulated genes in the two pathways also allow us to trace the synthesis source and flow destination of glutamate and arginine in *B*. *amyloliquefaciens*, such that production of L-glutamate must derive at least in part from L-histidine and further originate from phosphoribosyl pyrophosphate. Further, we can reason that glutamate and arginine here probably serve not only for the urea cycle but also as substrates for energy generation. For glutamate, this process seems to be completed by firstly converting L-glutamate to L-proline and then directing proline to TCA via pyruvate. Alternatively, glutamate could be converted into ornithine of urea cycle and integrated into TCA cycle via fumarate, in the course of which NADH were generated for ATP production.

Another example is the enrichment in the pathway of inositol phosphate metabolism. This enrichment suggested that at MSP catabolism of 1D-*myo*-inositol was finally directed into TCA cycle (S11 Fig). Inositol phosphates are abundant in most soils and the *myo* isomer is the most prevalent form occurring in nature [77]. Thus, we inferred that soil extract used in both SE and RS conditions supplied inositol phosphates, which were firstly transformed into *myo*-inositol, to serves as an important energy source at MSP. Besides, we also identified an enrichment of some repressed genes at #9h in the pathway relating to fructose and mannose metabolism (S11 Fig). Most of the genes were involved in the first part, before production of glyceraldehyde-3-phosphate, of glycolysis pathway. The suppression of these genes was probably resulted from the absence of easily decomposed saccharides at MSP, which may also account for inositol phosphate and amino acids were utilized as energy resource as deduced above. Overall, a number of regulated genes in the exemplified pathways display, at least partially, a profile of the potential surviving strategies of FZB42 adapting to rhizosphere.

Although the functions of most identified sRNAs yet to be understood, surging amount of evidences indicate their roles in bacterial adapation to changing environments and growth conditions [61, 78, 79]. For example, Spot42, one of the first sRNAs identified in *E*. *coli*, was recently shown to facilitate the metabolism reprogramming when switching to different carbon sources [78]; and the CsrB family of sRNAs regulate the expression of a large number of genes in response to altered nutrient availability [80–83]. Beyond central metabolism, sRNAs are now known involved in many bacterial life styles, including the switch between aerobic to anaerobic growth [84, 85], motility to biofilm formation [86], low population density to high density [87, 88], and saprophytism to virulence [21, 89, 90]. To make a living in the complex rhizosphere environments, bacteria need to possess a potential to utilize plant-derived nutrients, deal with dynamic biotic and abiotic challenges in the niches, colonize plant roots and form biofilm. Similar to the environmental alteration confronted by pathogenic bacteria during the course of infection, it is reasonable to expect that the identified *Bacillus* regulatory RNAs may also coordinate rhizobacterial adaptation to rhizosphere.

Here we have identified a large number of non-coding regulatory RNAs in FZB42. Specifically, we annotated in the FZB42 genome 53 *cis*-encoded riboswitch or leader sequence, 136 asRNAs, and 86 sRNA candidates, a number of which have been validated by independent northern blotting method. Some of the candidates could not be confirmed by independent northern blot analysis probably due to lower expression level and/or lower sensitivity of compared to RNA-seq. The validated sRNAs assemble an array of novel regulators in *Bacillus* worthy of further scrutiny. From their gene locations, most of the sRNAs are probably *trans*-acting sRNAs and act on genes from a distant locus. These sRNAs often directly binds to the 5’UTR of the target mRNAs to modulate translation initiation [61]. Importantly, our global TSS map has enabled a genome-wide annotation of 5’UTRs in FZB42, and this information would be immersely valuable for styding sRNA regulations, identifying their target sites in mRNAs, and establishing sRNA-mRNA interaction networks in the long term.

In summary, we have systemically identified the TSSs and non-coding RNAs of the rhizobacterium FZB42 in this study. Our results provide a valuable resource for gene studies on *B*. *amyloliquefaciens* and related species, *e*.*g*., the model organism *B*. *subtilis*. Detection of sRNAs in rhizobacteria not only broadens the scope of sRNA world in the model Gram positive rhizobacteria, but also helps to elucidate the functional insights into sRNAs in some unexplored environmental conditions, *e*.*g*., rhizosphere, and in some important physiological behavior, *e*.*g*., sporulation.

## Supporting Information

S1 Fig Functional classification of the genes up-regulated at middle stationary phase. The genes specifically up-regulated at middle stationary phase compared to early stationary phase were identified for SE samples and RS samples respectively. The common genes exhibited in both of the two conditions were distributed in various functional classes according to [91]. The inner pie map was the major class while the outer pie map were subclass subordinated to each major group. The percentage and/or number of the genes in each class/subclass are indicated respectively.
(PDF)

S2 Fig Sequencing reads of the *yhaA-yhfA-yhgC* operon. Two operons identified antisense to a known operon (A) or to known genes (B). cDNA reads in each library without (-TEX) or with (+TEX) terminator exonuclease treatment were mapped to *B*. *amyloliquefaciens* FZB42 chromosome and visualized by IGB 8.1. Vertical bars indicate cDNA recovery detected in each library which were adjusted to the same scale of 20 or 30. The genes indicated above the horizontal coordinates are located on the forward strand while the genes indicated below the horizontal coordinates are located on the reverse strand. Bacterial cultures were sampled for total RNA extraction at two time points (#6h&#9h) from four conditions: i) in 1CM medium; ii) 1CM medium supplemented with the maize root exudates (+RE); iii) 1CM medium supplemented with soil extract (+SE); iv) 1CM medium supplemented with both maize root exudates and soil extract (+RS). The time point #6h corresponded to early stationary phase while #9h corresponded to middle stationary phase.
(PDF)

S3 Fig Sequencing reads of the transcript antisense to *yndL*. cDNA reads in each library without (-TEX) or with (+TEX) terminator exonuclease treatment were mapped to *B*. *amyloliquefaciens* FZB42 chromosome and visualized by IGB 8.1. Vertical bars indicate cDNA recovery detected in each library which were adjusted to the same scale of 50. The genes indicated above the horizontal coordinates are located on the forward strand while the genes indicated below the horizontal coordinates are located on the reverse strand. Bacterial cultures were sampled for total RNA extraction at two time points (#6h&#9h) from four conditions: i) in 1CM medium; ii) 1CM medium supplemented with the maize root exudates (+RE); iii) 1CM medium supplemented with soil extract (+SE); iv) 1CM medium supplemented with both maize root exudates and soil extract (+RS). The time point #6h corresponded to early stationary phase while #9h corresponded to middle stationary phase.
(PDF)

S4 Fig Expression of sRNA Bas28 (*rhaX-yheI*) shown in Northern Blot and RNA-seq result. Panel A: Northern blot was performed using total RNAs prepared from different sampling time points and different media. The arrow indicates the position where the sRNA was expected to be present according to RNA-seq result. Panel B: cDNA reads in each library without (-TEX) or with (+TEX) terminator exonuclease treatment were mapped to *B*. *amyloliquefaciens* FZB42 chromosome and visualized by IGB 8.1. Vertical bars indicate cDNA recovery detected in each library which were adjusted to the same scale of 50. The genes indicated above the horizontal coordinates are located on the forward strand while the genes indicated below the horizontal coordinates are located on the reverse strand. Bacterial cultures were sampled for total RNA extraction at two time points (#6h&#9h) from four media: i) in 1CM medium; ii) 1CM medium supplemented with the maize root exudates (+RE); iii) 1CM medium supplemented with soil extract (+SE); iv) 1CM medium supplemented with both maize root exudates and soil extract (+RS). The time point #6h corresponded to early stationary phase while #9h corresponded to middle stationary phase.
(PDF)

S5 Fig Expression of sRNA Bas35 (*trpS-oppA*) shown in RNA-seq result and Northern Blot. Panel A: Northern blot was performed using total RNAs prepared from different sampling time points and different media. The arrow indicates the position where the sRNA was expected to be present according to RNA-seq result. Panel B: cDNA reads in each library without (-TEX) or with (+TEX) terminator exonuclease treatment were mapped to *B*. *amyloliquefaciens* FZB42 chromosome and visualized by IGB 8.1. Vertical bars indicate cDNA recovery detected in each library which were adjusted to the same scale of 50. The genes indicated above the horizontal coordinates are located on the forward strand while the genes indicated below the horizontal coordinates are located on the reverse strand. Bacterial cultures were sampled for total RNA extraction at two time points (#6h&#9h) from four media: i) in 1CM medium; ii) 1CM medium supplemented with the maize root exudates (+RE); iii) 1CM medium supplemented with soil extract (+SE); iv) 1CM medium supplemented with both maize root exudates and soil extract (+RS). The time point #6h corresponded to early stationary phase while #9h corresponded to middle stationary phase.
(PDF)

S6 Fig Expression of the verified sRNAs along growth of FZB42 in four different media. Bacterial cultures were sampled for total RNA extraction at different time points from four media: i) in 1CM medium; ii) 1CM medium supplemented with the maize root exudates (+RE); iii) 1CM medium supplemented with soil extract (+SE); iv) 1CM medium supplemented with both maize root exudates and soil extract (+RS). Northern blots were performed using corresponding oligo probes for each sRNA (S16 Table). In panel A and panel B, the arrows and the numbers at the end of each arrow indicate time points where cultures were collected. The numbers of RNA samples used for Northern blot in panel C & D corresponded to the culture numbers in panel A, while the numbers of RNA samples used for Northern blot in panel E-H corresponded to culture numbers in panel B.
(PDF)

S7 Fig Expression of sRNA Bas26 (*yjcA-RBAM_011810*) shown in RNA-seq result. cDNA reads in each library without (-TEX) or with (+TEX) terminator exonuclease treatment were mapped to *B*. *amyloliquefaciens* FZB42 chromosome and visualized by IGB 8.1. Vertical bars indicate cDNA recovery detected in each library which were adjusted to the same scale of 50. The genes indicated above the horizontal coordinates are located on the forward strand while the genes indicated below the horizontal coordinates are located on the reverse strand. Bacterial cultures were sampled for total RNA extraction at two time points (#6h&#9h) from two conditions: i) 1CM medium supplemented with soil extract (+SE); ii) 1CM medium supplemented with maize root exudates and soil extract (+RS). The time point #6h corresponded to early stationary phase while #9h corresponded to middle stationary phase.
(PDF)

S8 Fig Sequencing reads of the genes involved in plant-microbe interaction. cDNA reads in each library without (-TEX) or with (+TEX) terminator exonuclease treatment were mapped to *B*. *amyloliquefaciens* FZB42 chromosome and visualized by IGB 8.1. Vertical bars indicate cDNA recovery detected in each library which were adjusted to the same scale of 50. The genes indicated above the horizontal coordinates are located on the forward strand while the genes indicated below the horizontal coordinates are located on the reverse strand. Bacterial cultures were sampled for total RNA extraction at two time points (#6h&#9h) from four conditions: i) in 1CM medium; ii) 1CM medium supplemented with the maize root exudates (+RE); iii) 1CM medium supplemented with soil extract (+SE); iv) 1CM medium supplemented with both maize root exudates and soil extract (+RS). The time point #6h corresponded to early stationary phase while #9h corresponded to middle stationary phase.
(PDF)

S9 Fig Expression of yqzM (*holA-comEC*) shown in RNA-seq result. cDNA reads in each library without (-TEX) or with (+TEX) terminator exonuclease treatment were mapped to *B*. *amyloliquefaciens* FZB42 chromosome and visualized by IGB 8.1. Vertical bars indicate cDNA recovery detected in each library which were adjusted to the same scale of 50. The genes indicated above the horizontal coordinates are located on the forward strand while the genes indicated below the horizontal coordinates are located on the reverse strand. Bacterial cultures were sampled for total RNA extraction at two time points (#6h&#9h) from four conditions: i) in 1CM medium; ii) 1CM medium supplemented with the maize root exudates (+RE); iii) 1CM medium supplemented with soil extract (+SE); iv) 1CM medium supplemented with both maize root exudates and soil extract (+RS). The time point #6h corresponded to early stationary phase while #9h corresponded to middle stationary phase.
(PDF)

S10 Fig Expressed transcripts occurred in the region of locus *dps-ytkA*. cDNA reads in each library without (-TEX) or with (+TEX) terminator exonuclease treatment were mapped to *B*. *amyloliquefaciens* FZB42 chromosome and visualized by IGB 8.1. Vertical bars indicate cDNA recovery detected in each library which were adjusted to the same scale of 50. The genes indicated above the horizontal coordinates are located on the forward strand while the genes indicated below the horizontal coordinates are located on the reverse strand. Bacterial cultures were sampled for total RNA extraction at two time points (#6h&#9h) from four conditions: i) in 1CM medium; ii) 1CM medium supplemented with the maize root exudates (+RE); iii) 1CM medium supplemented with soil extract (+SE); iv) 1CM medium supplemented with both maize root exudates and soil extract (+RS). The time point #6h corresponded to early stationary phase while #9h corresponded to middle stationary phase
(PDF)

S11 Fig A group of significantly regulated genes in #9h samples compared with in #6h samples are known in function involved in histidine metabolism (A), arginine and proline metabolism (B), inositol phosphate metabolism (C), and fructose and mannose metabolism (D). The regulated genes were mapped in the KEGG pathway and the diagram was accordingly adapted. The products encoded by up-regulated genes at middle stationary phase are highlighted in red while those by down-regulated genes are highlighted in green. RE: *B*. *amyloliquefaciens* FZB42 was grown in 1CM medium supplemented with the maize root exudates (RE); RS: *B*. *amyloliquefaciens* FZB42 was grown in 1CM medium supplemented with the maize root exudates (RE) and soil extract (SE).
(PDF)

S1 Table Growth conditions and distribution of sequencing reads mapped to *B*. *amyloliquefaciens* FZB42 genome in each library. (XLSX)

S2 Table Statistics of number and percentage of all categories TSSs detected in various growth condition. (XLSX)

S3 Table The 10 primary TSSs randomly selected to check robustness of TSSs identification. (XLSX)

S4 Table Primary TSSs specific to one growth condition. (XLSX)

S5 Table Leaderless mRNAs detected in *B*. *amyloliquefaciens* FZB42. (XLSX)

S6 Table Secondary TSSs specific to middle stationary phase (SE#9h and RS#9h). (XLSX)

S7 Table The significantly regulated mRNA genes by different growth phase. (XLSX)

S8 Table Cis-encoded regulatory RNAs identified. (XLSX)

S9 Table RNAs antisense to the middle region of a gene. (XLSX)

S10 Table Antisense RNAs locating at an intergenic region. (XLSX)

S11 Table Regulatory RNAs highly expressed at middle stationary phase. (XLSX)

S12 Table Re-annotation of mRNA genes. (XLSX)

S13 Table Annotation of tRNAs and mRNA genes newly identified. (XLSX)

S14 Table Predicted operons in genome of *B*. *amyloliquefaciens* FZB42. (XLSX)

S15 Table The genes expressed from an inner TSS within a polycistronic operon. (XLSX)

S16 Table Small RNAs detected by Northern Blot. (XLSX)

S17 Table Genes involved in plant-bacteria interaction. (XLSX)
