# Supplementary figures and images for: dRNA-Seq Reveals Genomewide TSSs and Noncoding RNAs of Plant Beneficial Rhizobacterium Bacillus amyloliquefaciens FZB42
Source: PLoS One. 2015 Nov 5;10(11):e0142002. doi: 10.1371/journal.pone.0142002 (PMC4634765; doi:10.1371/journal.pone.0142002)

Fan et al, 2015

Suppl. Fig. 1

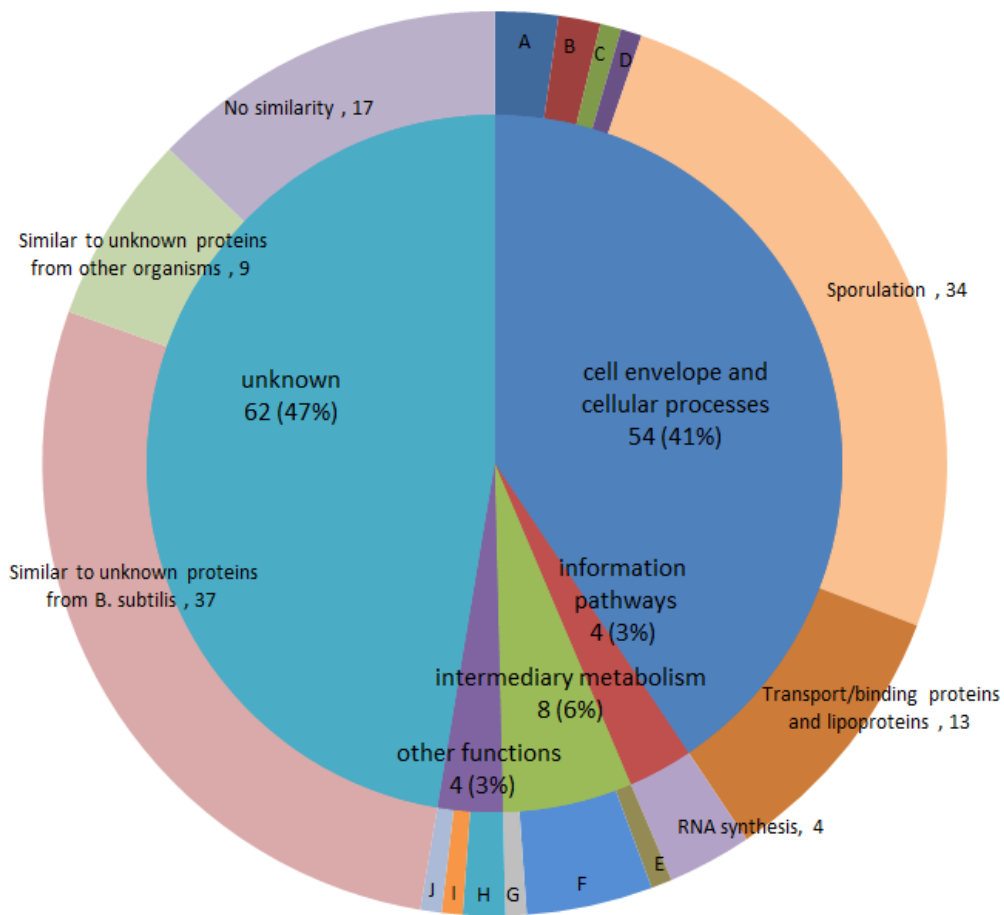

Supplement: S1 Fig — The genes specifically up-regulated at middle stationary phase compared to early stationary phase were identified for SE samples and RS samples respectively. The common genes exhibited in both of the two conditions were distributed in various functional classes according to [91]. The inner pie map was the major class while the outer pie map were subclass subordinated to each major group. The percentage and/or number of the genes in each class/subclass are indicated respectively. (PDF) [file pone.0142002.s001.pdf]

# Fan et al, 2015

## Suppl. Fig. 2

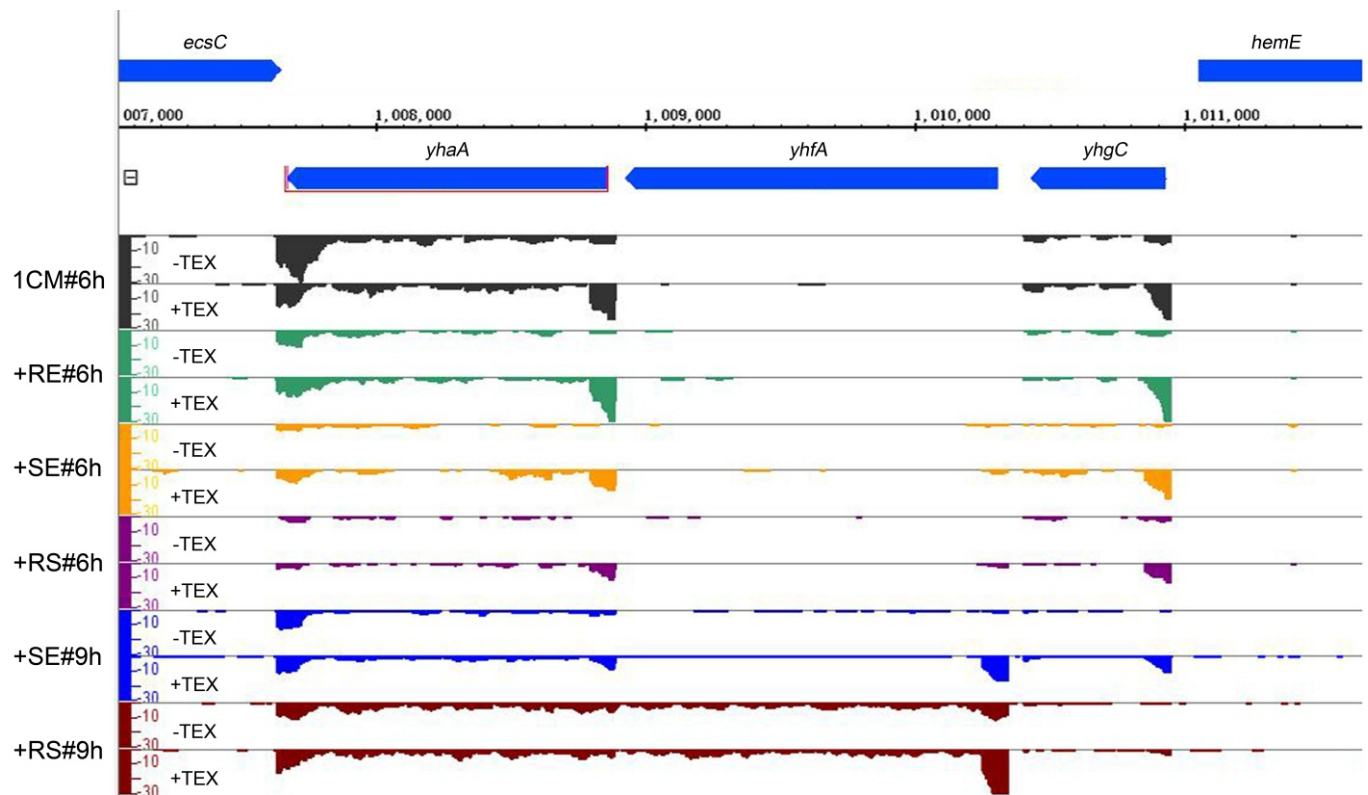

Supplement: S2 Fig — Two operons identified antisense to a known operon (A) or to known genes (B). cDNA reads in each library without (-TEX) or with (+TEX) terminator exonuclease treatment were mapped to B. amyloliquefaciens FZB42 chromosome and visualized by IGB 8.1. Vertical bars indicate cDNA recovery detected in each library which were adjusted to the same scale of 20 or 30. The genes indicated above the horizontal coordinates are located on the forward strand while the genes indicated below the horizontal coordinates are located on the reverse strand. Bacterial cultures were sampled for total RNA extraction at two time points (#6h h) from four conditions: i) in 1CM medium; ii) 1CM medium supplemented with the maize root exudates (+RE); iii) 1CM medium supplemented with soil extract (+SE); iv) 1CM medium supplemented with both maize root exudates and soil extract (+RS). The time point #6h corresponded to early stationary phase while #9h corresponded to middle stationary phase. (PDF) [file pone.0142002.s002.pdf]

Fan et al, 2015  
Suppl. Fig. 3

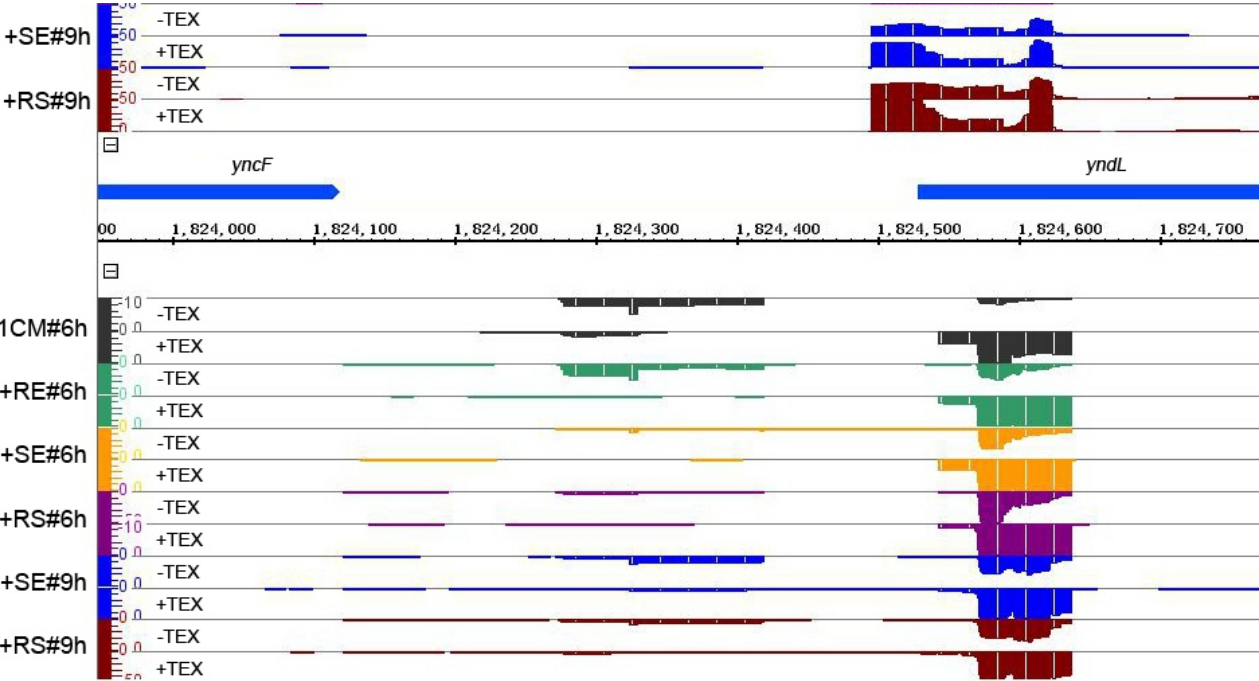

Supplement: S3 Fig — cDNA reads in each library without (-TEX) or with (+TEX) terminator exonuclease treatment were mapped to B. amyloliquefaciens FZB42 chromosome and visualized by IGB 8.1. Vertical bars indicate cDNA recovery detected in each library which were adjusted to the same scale of 50. The genes indicated above the horizontal coordinates are located on the forward strand while the genes indicated below the horizontal coordinates are located on the reverse strand. Bacterial cultures were sampled for total RNA extraction at two time points (#6h h) from four conditions: i) in 1CM medium; ii) 1CM medium supplemented with the maize root exudates (+RE); iii) 1CM medium supplemented with soil extract (+SE); iv) 1CM medium supplemented with both maize root exudates and soil extract (+RS). The time point #6h corresponded to early stationary phase while #9h corresponded to middle stationary phase. (PDF) [file pone.0142002.s003.pdf]

Fan et al, 2015  
Suppl. Fig. 4

A

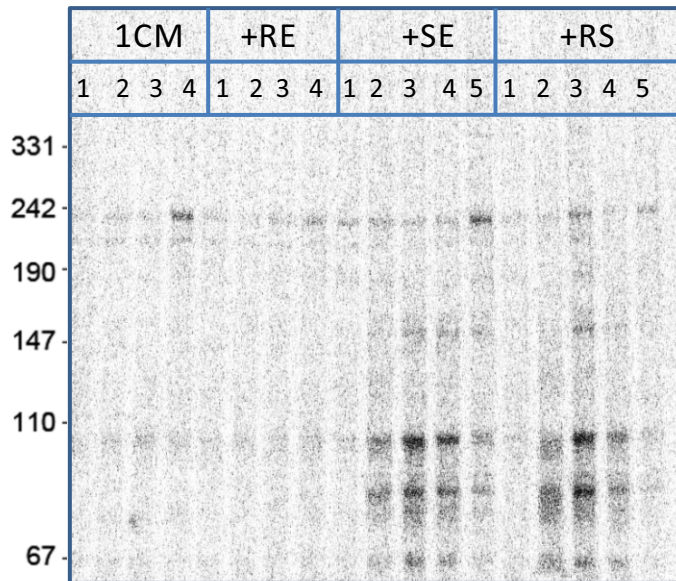

B

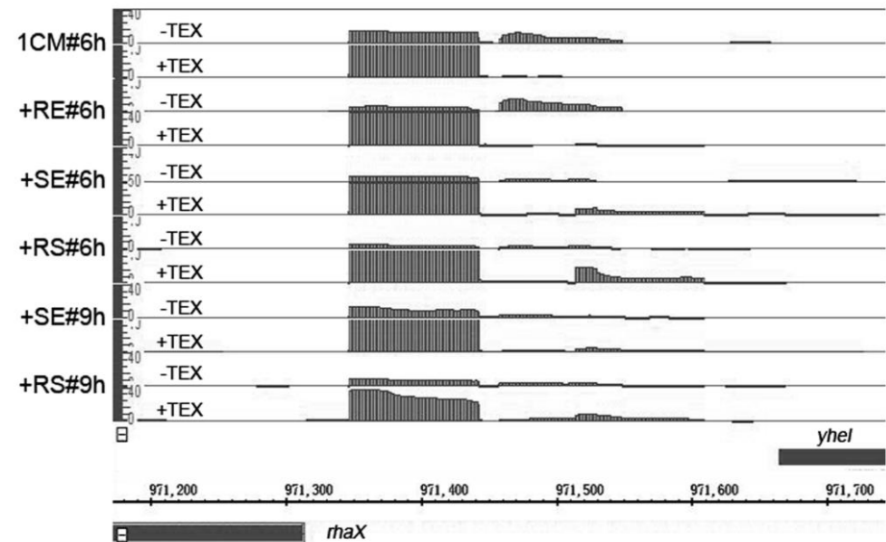

Supplement: S4 Fig — Panel A: Northern blot was performed using total RNAs prepared from different sampling time points and different media. The arrow indicates the position where the sRNA was expected to be present according to RNA-seq result. Panel B: cDNA reads in each library without (-TEX) or with (+TEX) terminator exonuclease treatment were mapped to B. amyloliquefaciens FZB42 chromosome and visualized by IGB 8.1. Vertical bars indicate cDNA recovery detected in each library which were adjusted to the same scale of 50. The genes indicated above the horizontal coordinates are located on the forward strand while the genes indicated below the horizontal coordinates are located on the reverse strand. Bacterial cultures were sampled for total RNA extraction at two time points (#6h h) from four media: i) in 1CM medium; ii) 1CM medium supplemented with the maize root exudates (+RE); iii) 1CM medium supplemented with soil extract (+SE); iv) 1CM medium supplemented with both maize root exudates and soil extract (+RS). The time point #6h corresponded to early stationary phase while #9h corresponded to middle stationary phase. (PDF) [file pone.0142002.s004.pdf]

Fan et al, 2015  
Suppl. Fig. 5

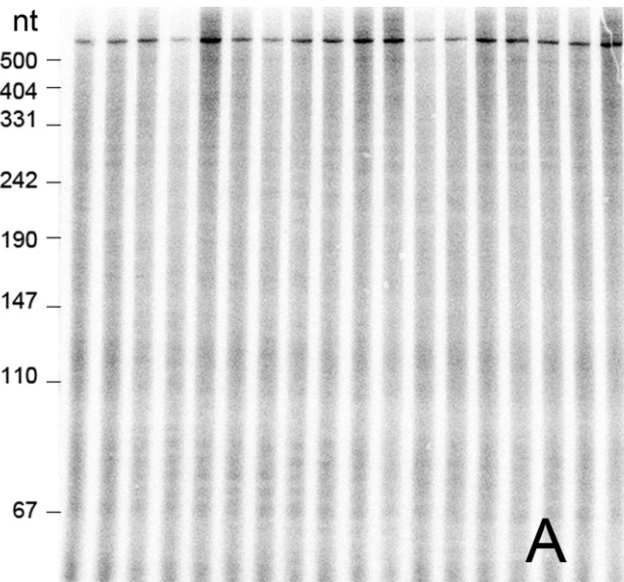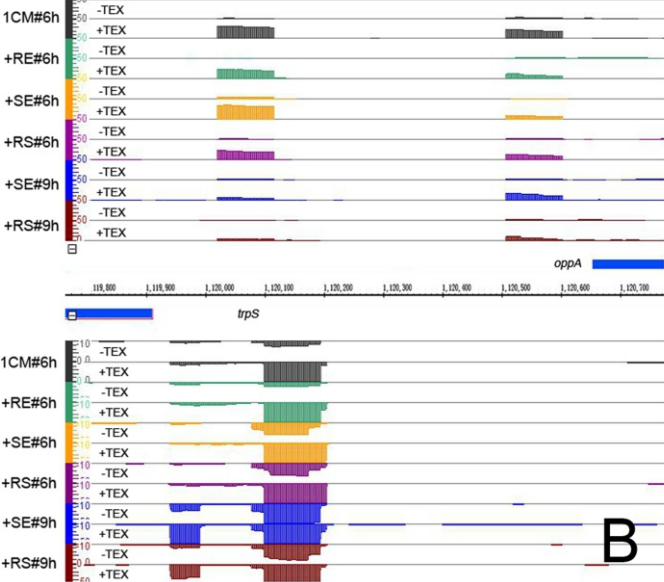

Supplement: S5 Fig — Panel A: Northern blot was performed using total RNAs prepared from different sampling time points and different media. The arrow indicates the position where the sRNA was expected to be present according to RNA-seq result. Panel B: cDNA reads in each library without (-TEX) or with (+TEX) terminator exonuclease treatment were mapped to B. amyloliquefaciens FZB42 chromosome and visualized by IGB 8.1. Vertical bars indicate cDNA recovery detected in each library which were adjusted to the same scale of 50. The genes indicated above the horizontal coordinates are located on the forward strand while the genes indicated below the horizontal coordinates are located on the reverse strand. Bacterial cultures were sampled for total RNA extraction at two time points (#6h h) from four media: i) in 1CM medium; ii) 1CM medium supplemented with the maize root exudates (+RE); iii) 1CM medium supplemented with soil extract (+SE); iv) 1CM medium supplemented with both maize root exudates and soil extract (+RS). The time point #6h corresponded to early stationary phase while #9h corresponded to middle stationary phase. (PDF) [file pone.0142002.s005.pdf]

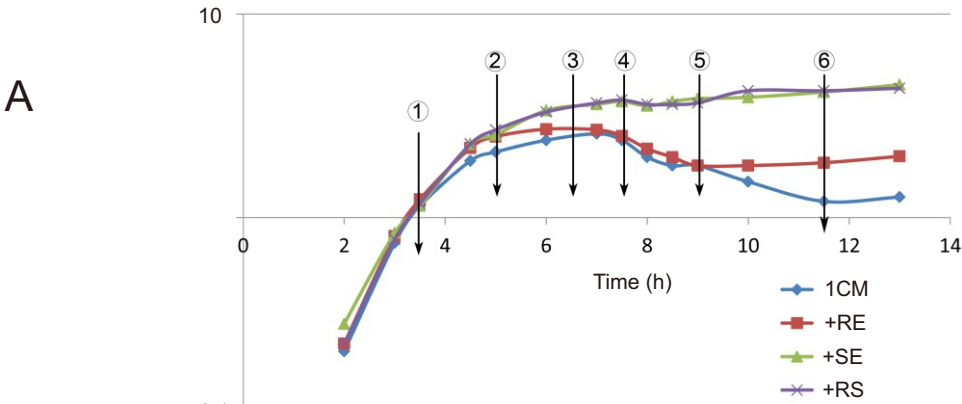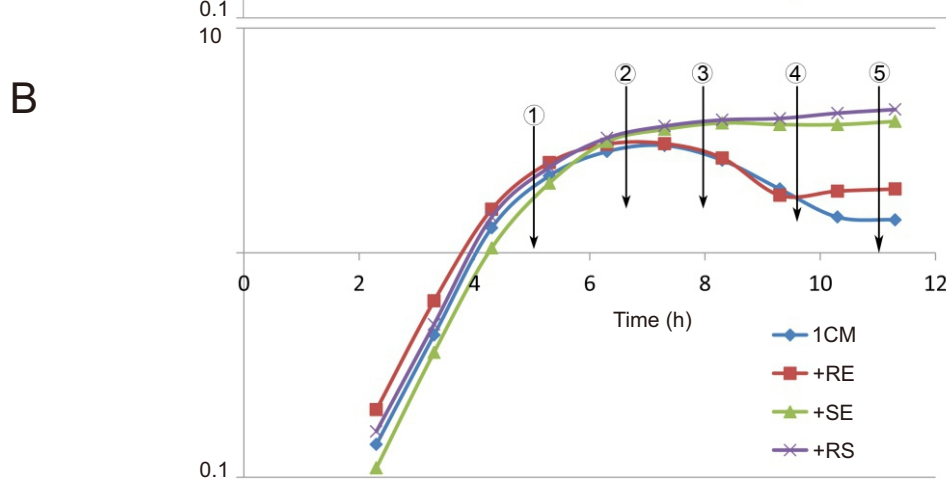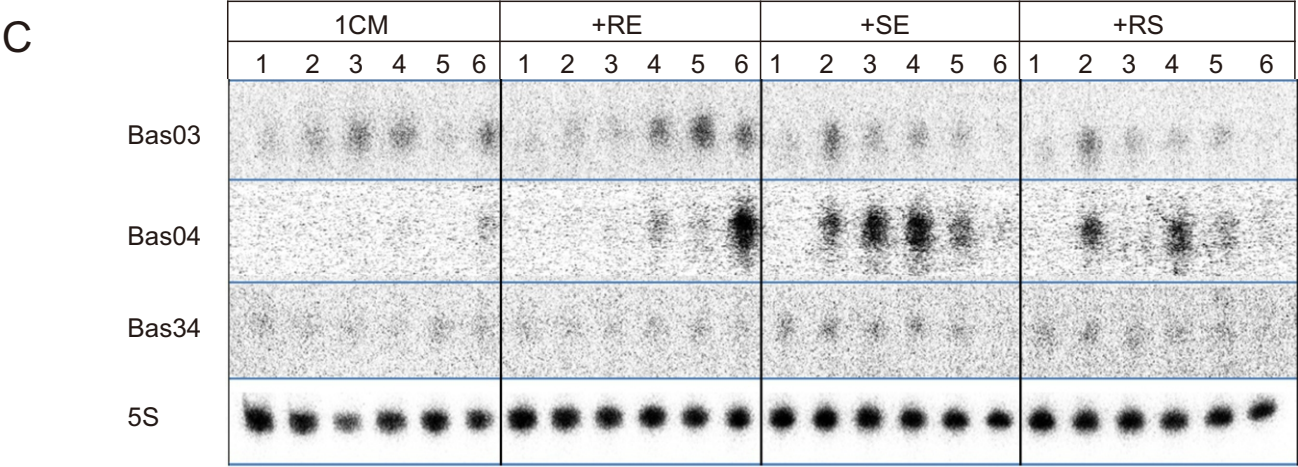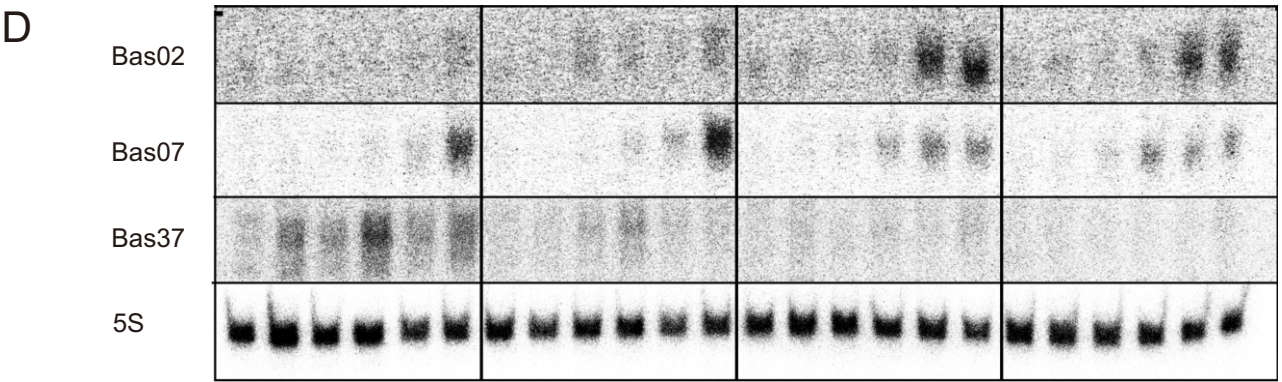

E

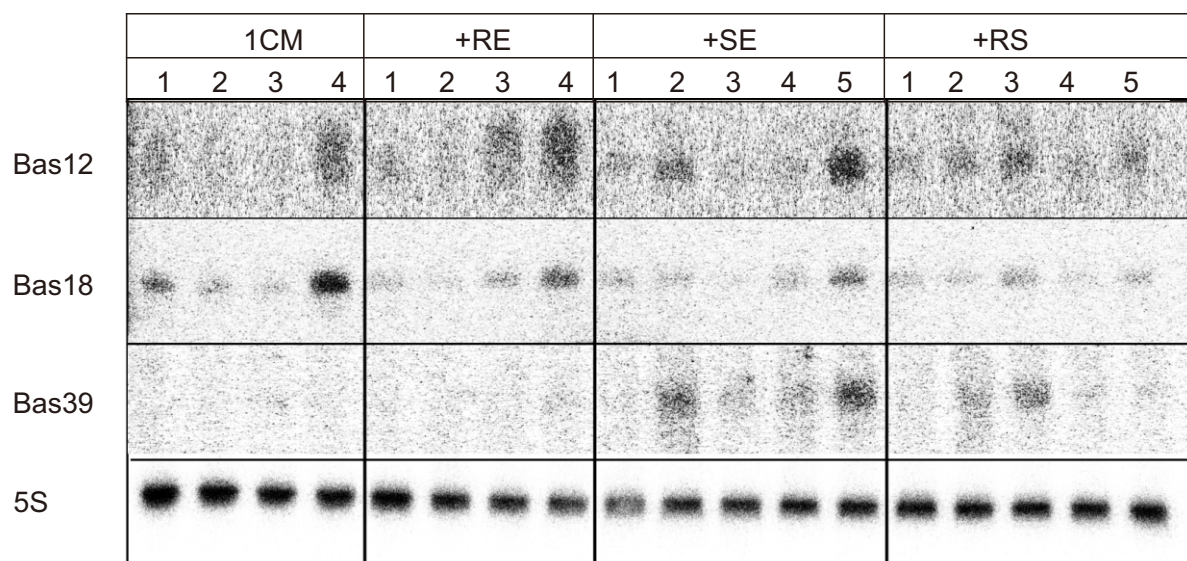

F

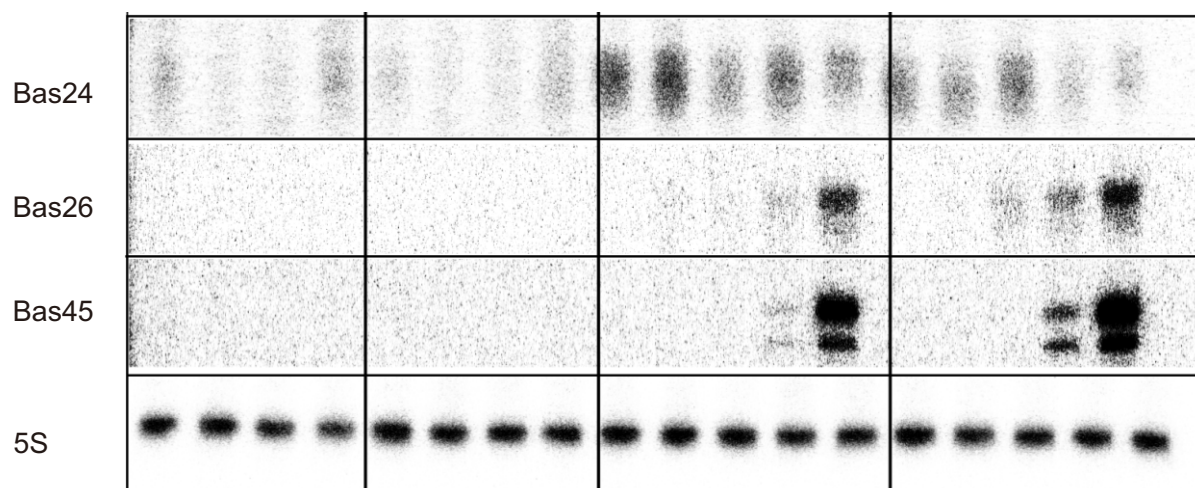

G

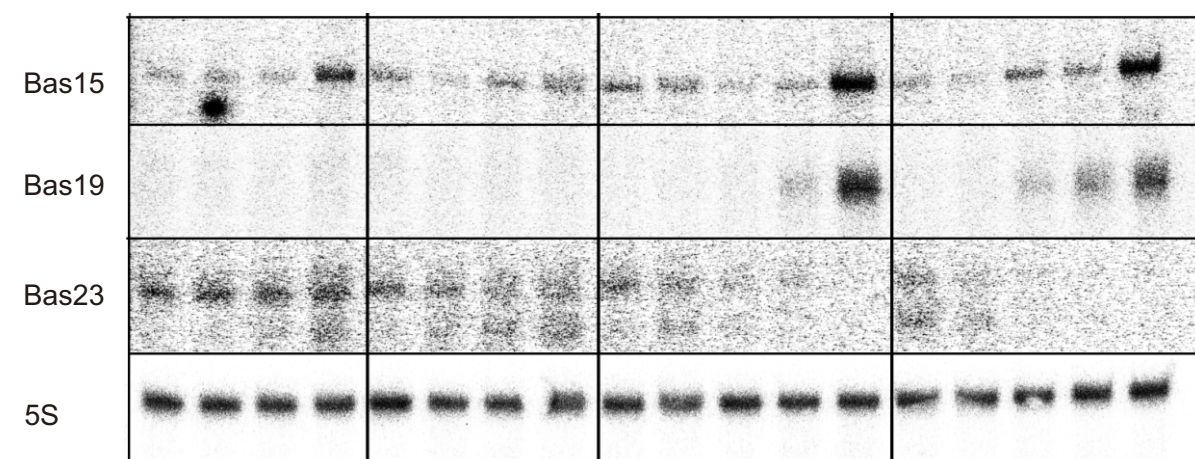

H

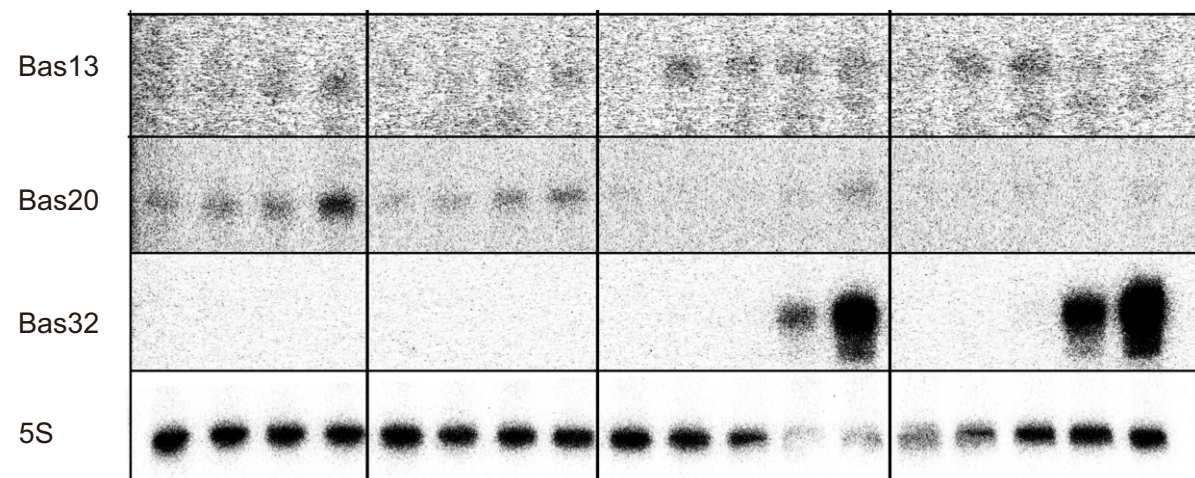

Supplement: S6 Fig — Bacterial cultures were sampled for total RNA extraction at different time points from four media: i) in 1CM medium; ii) 1CM medium supplemented with the maize root exudates (+RE); iii) 1CM medium supplemented with soil extract (+SE); iv) 1CM medium supplemented with both maize root exudates and soil extract (+RS). Northern blots were performed using corresponding oligo probes for each sRNA (S16 Table). In panel A and panel B, the arrows and the numbers at the end of each arrow indicate time points where cultures were collected. The numbers of RNA samples used for Northern blot in panel C & D corresponded to the culture numbers in panel A, while the numbers of RNA samples used for Northern blot in panel E-H corresponded to culture numbers in panel B. (PDF) [file pone.0142002.s006.pdf]

Fan et al, 2015  
Suppl. Fig. 7

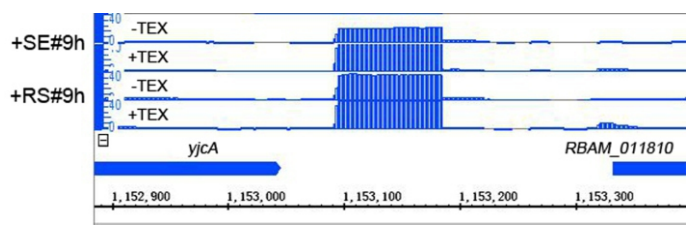

Supplement: S7 Fig — cDNA reads in each library without (-TEX) or with (+TEX) terminator exonuclease treatment were mapped to B. amyloliquefaciens FZB42 chromosome and visualized by IGB 8.1. Vertical bars indicate cDNA recovery detected in each library which were adjusted to the same scale of 50. The genes indicated above the horizontal coordinates are located on the forward strand while the genes indicated below the horizontal coordinates are located on the reverse strand. Bacterial cultures were sampled for total RNA extraction at two time points (#6h h) from two conditions: i) 1CM medium supplemented with soil extract (+SE); ii) 1CM medium supplemented with maize root exudates and soil extract (+RS). The time point #6h corresponded to early stationary phase while #9h corresponded to middle stationary phase. (PDF) [file pone.0142002.s007.pdf]

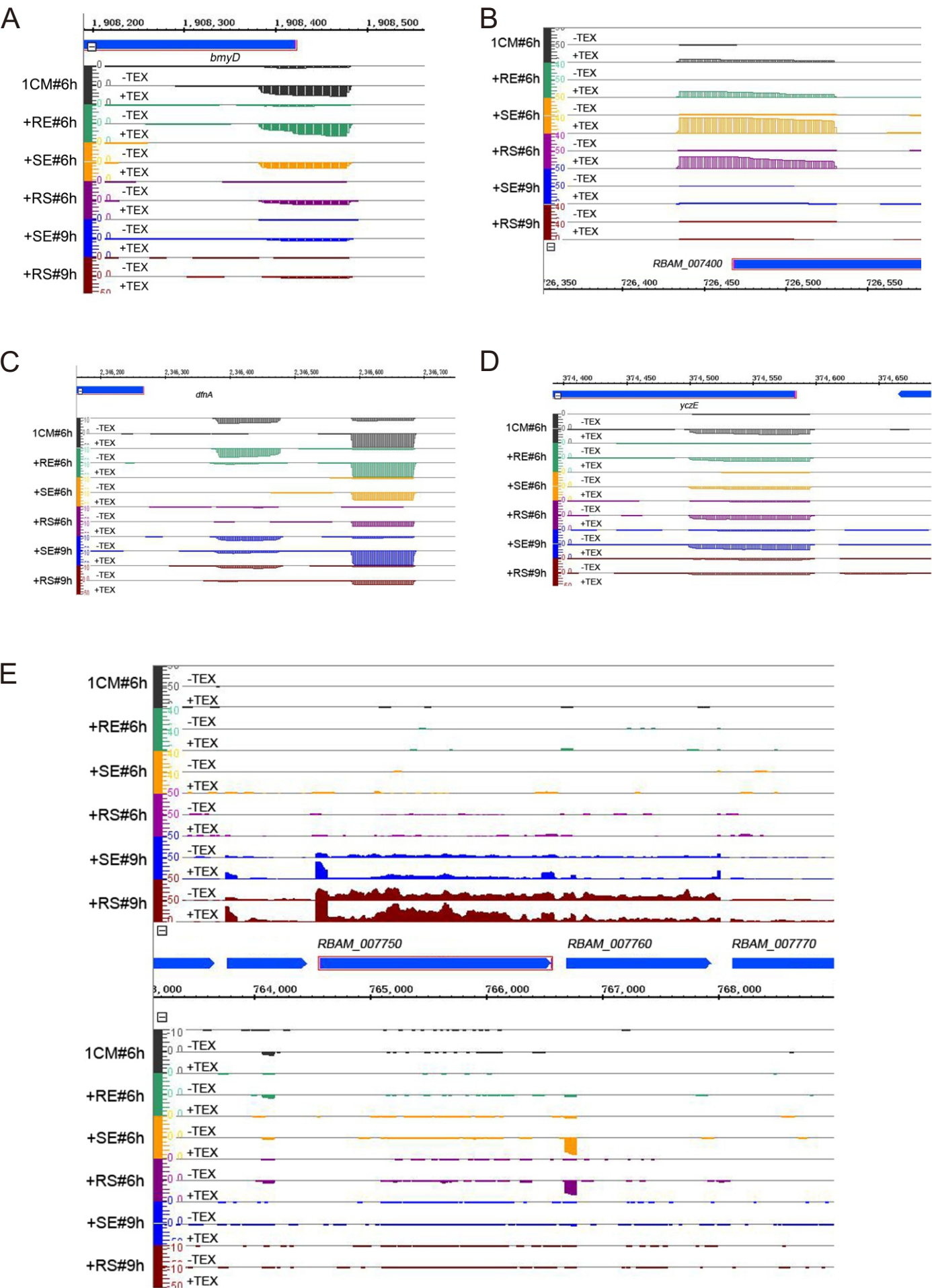

Supplement: S8 Fig — cDNA reads in each library without (-TEX) or with (+TEX) terminator exonuclease treatment were mapped to B. amyloliquefaciens FZB42 chromosome and visualized by IGB 8.1. Vertical bars indicate cDNA recovery detected in each library which were adjusted to the same scale of 50. The genes indicated above the horizontal coordinates are located on the forward strand while the genes indicated below the horizontal coordinates are located on the reverse strand. Bacterial cultures were sampled for total RNA extraction at two time points (#6h h) from four conditions: i) in 1CM medium; ii) 1CM medium supplemented with the maize root exudates (+RE); iii) 1CM medium supplemented with soil extract (+SE); iv) 1CM medium supplemented with both maize root exudates and soil extract (+RS). The time point #6h corresponded to early stationary phase while #9h corresponded to middle stationary phase. (PDF) [file pone.0142002.s008.pdf]

Fan et al, 2015  
Suppl. Fig. 9

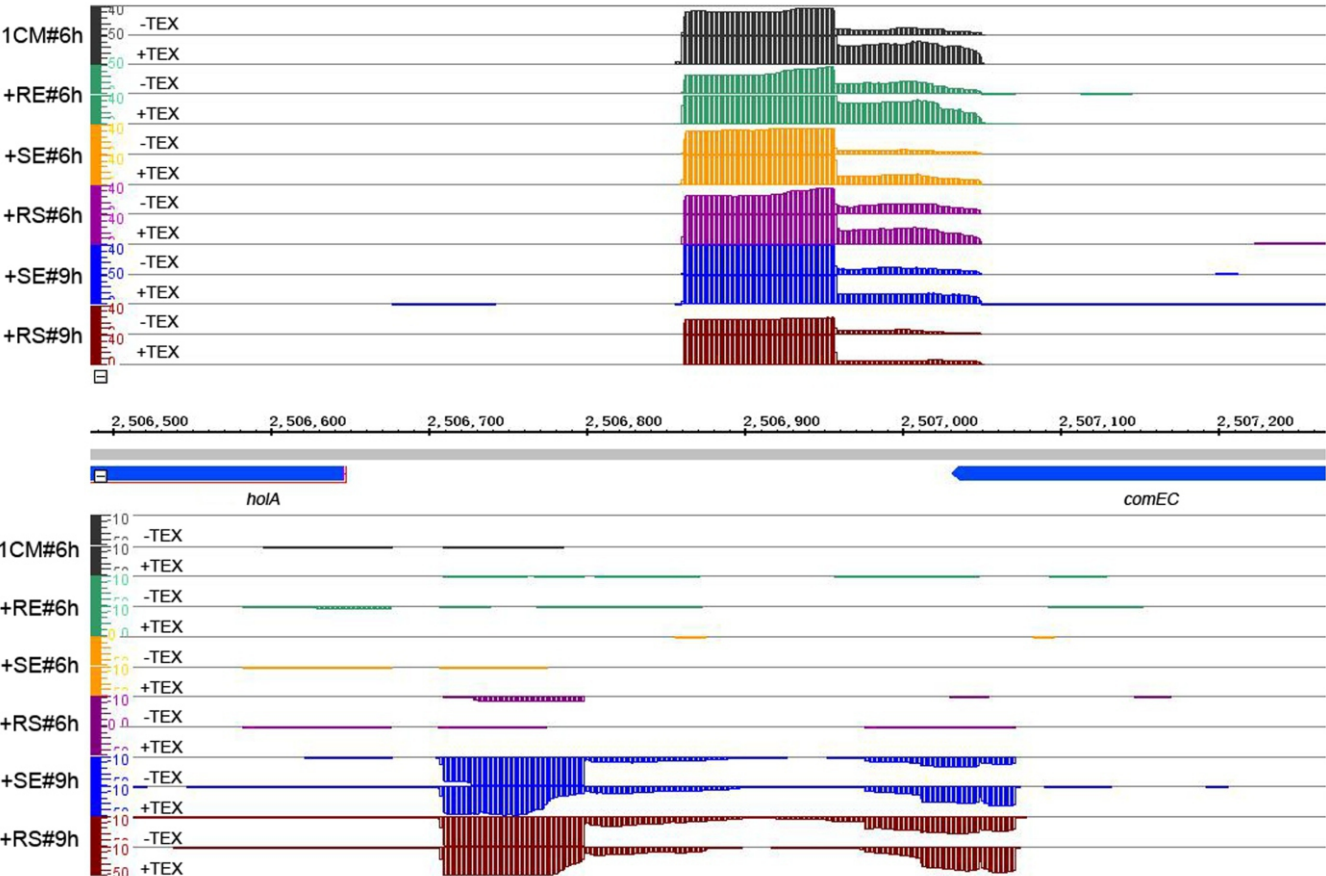

Supplement: S9 Fig — cDNA reads in each library without (-TEX) or with (+TEX) terminator exonuclease treatment were mapped to B. amyloliquefaciens FZB42 chromosome and visualized by IGB 8.1. Vertical bars indicate cDNA recovery detected in each library which were adjusted to the same scale of 50. The genes indicated above the horizontal coordinates are located on the forward strand while the genes indicated below the horizontal coordinates are located on the reverse strand. Bacterial cultures were sampled for total RNA extraction at two time points (#6h h) from four conditions: i) in 1CM medium; ii) 1CM medium supplemented with the maize root exudates (+RE); iii) 1CM medium supplemented with soil extract (+SE); iv) 1CM medium supplemented with both maize root exudates and soil extract (+RS). The time point #6h corresponded to early stationary phase while #9h corresponded to middle stationary phase. (PDF) [file pone.0142002.s009.pdf]

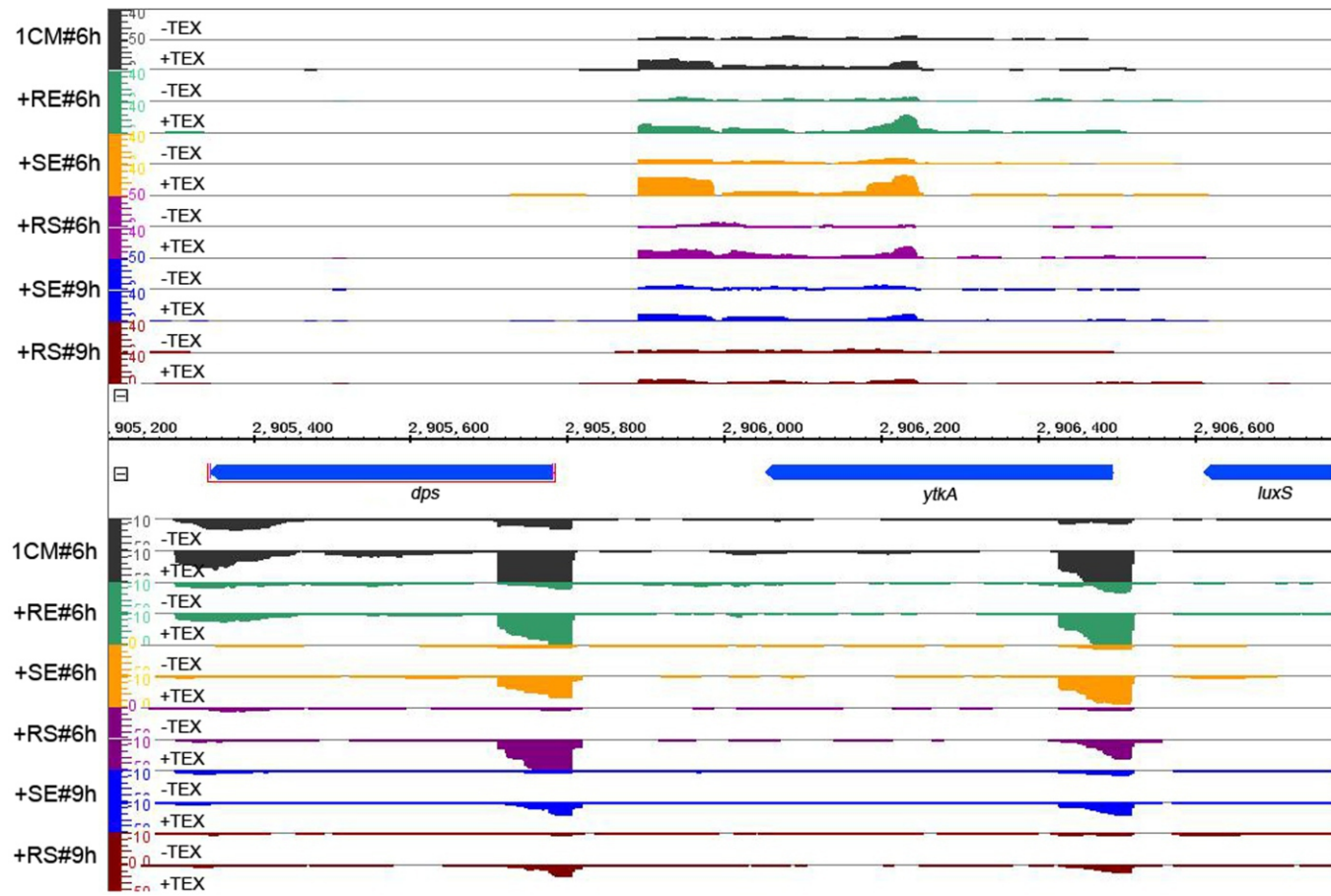

Supplement: S10 Fig — cDNA reads in each library without (-TEX) or with (+TEX) terminator exonuclease treatment were mapped to B. amyloliquefaciens FZB42 chromosome and visualized by IGB 8.1. Vertical bars indicate cDNA recovery detected in each library which were adjusted to the same scale of 50. The genes indicated above the horizontal coordinates are located on the forward strand while the genes indicated below the horizontal coordinates are located on the reverse strand. Bacterial cultures were sampled for total RNA extraction at two time points (#6h h) from four conditions: i) in 1CM medium; ii) 1CM medium supplemented with the maize root exudates (+RE); iii) 1CM medium supplemented with soil extract (+SE); iv) 1CM medium supplemented with both maize root exudates and soil extract (+RS). The time point #6h corresponded to early stationary phase while #9h corresponded to middle stationary phase (PDF) [file pone.0142002.s010.pdf]

Data on KEGG graph  
Rendered by Pathview

Data on KEGG graph  
Rendered by Pathview

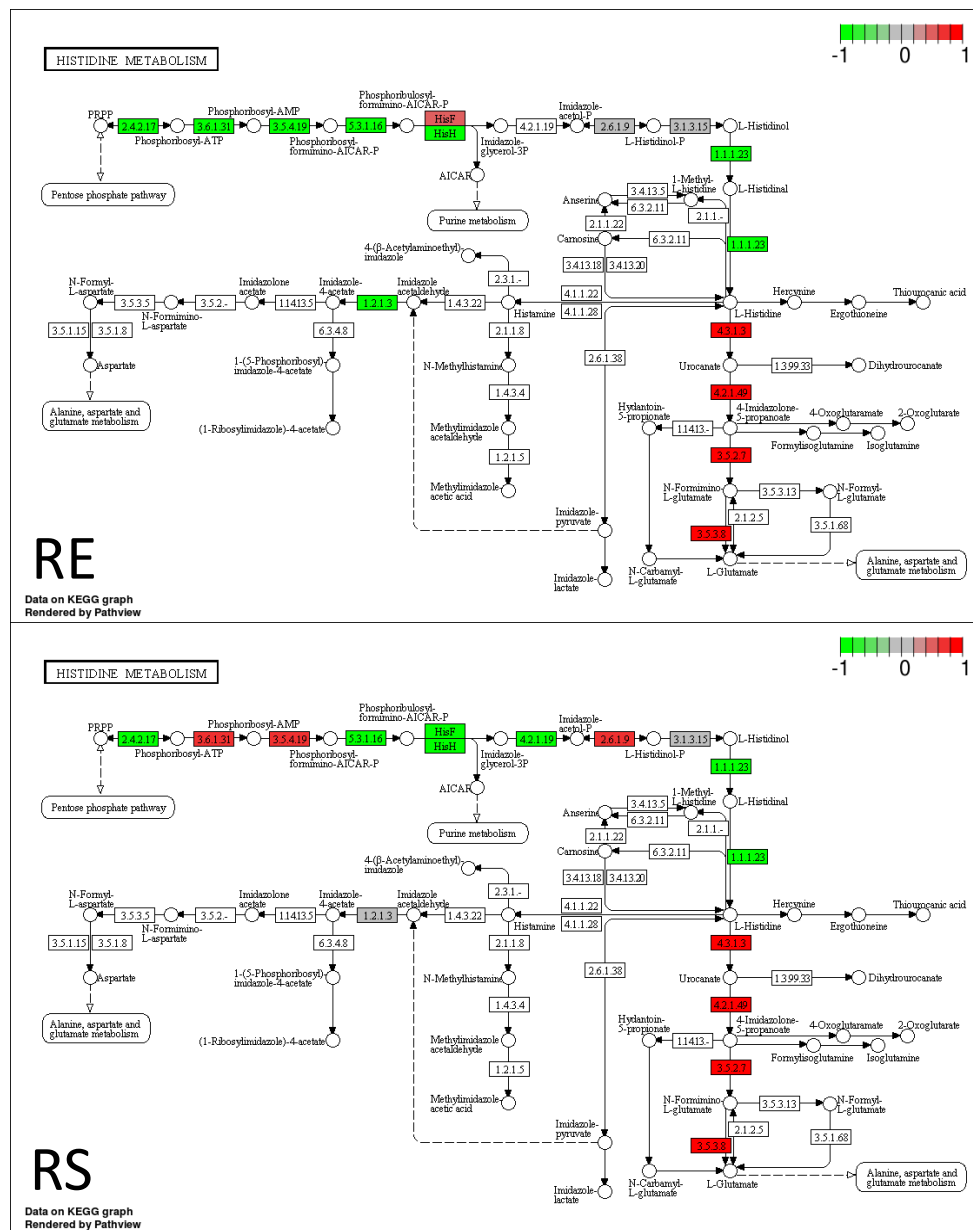

# B

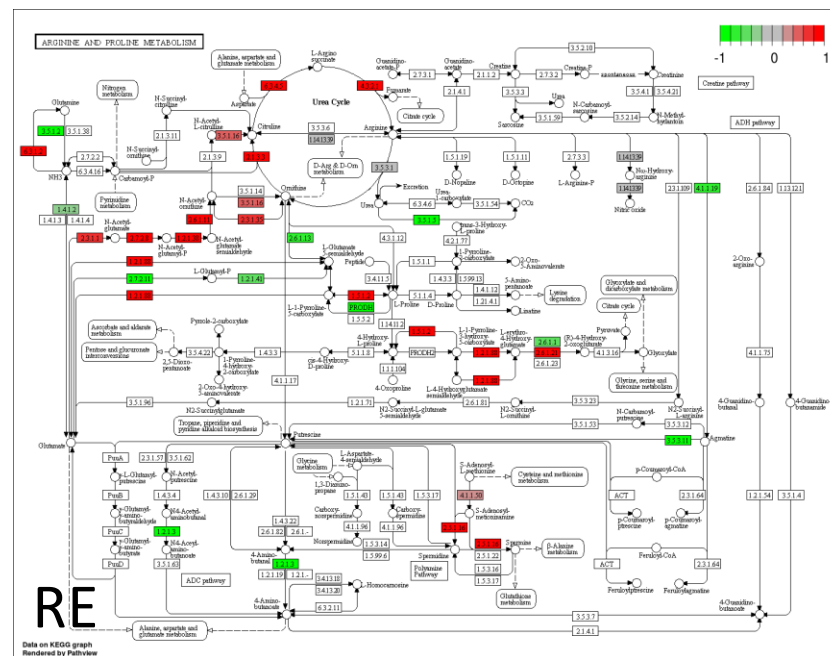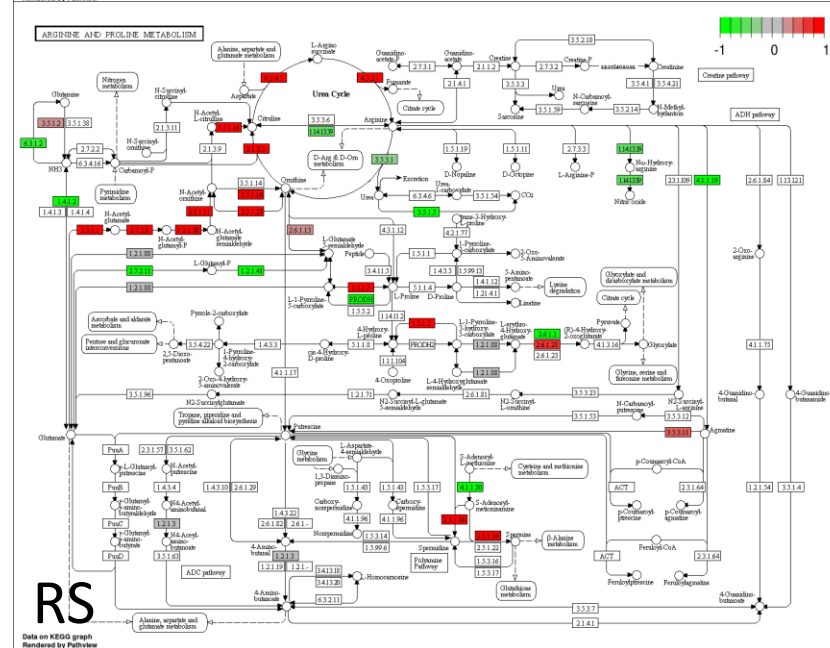

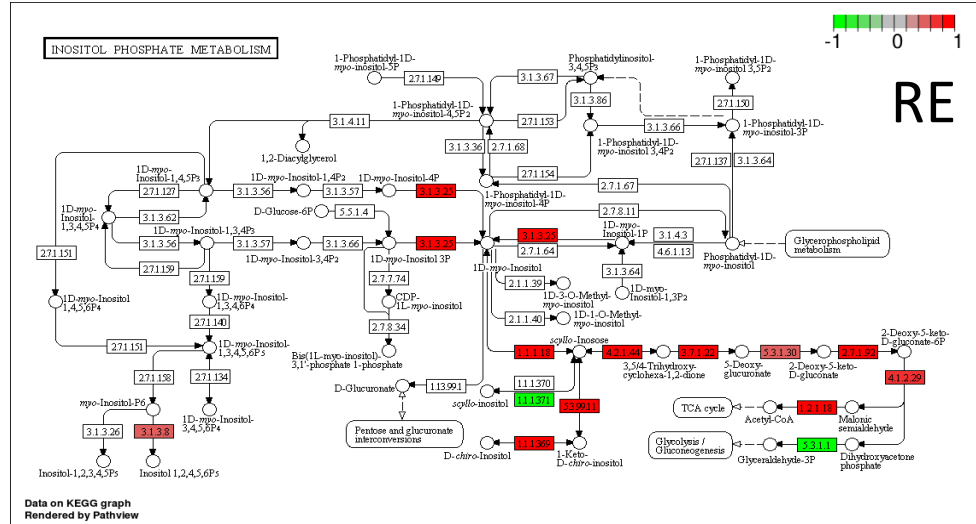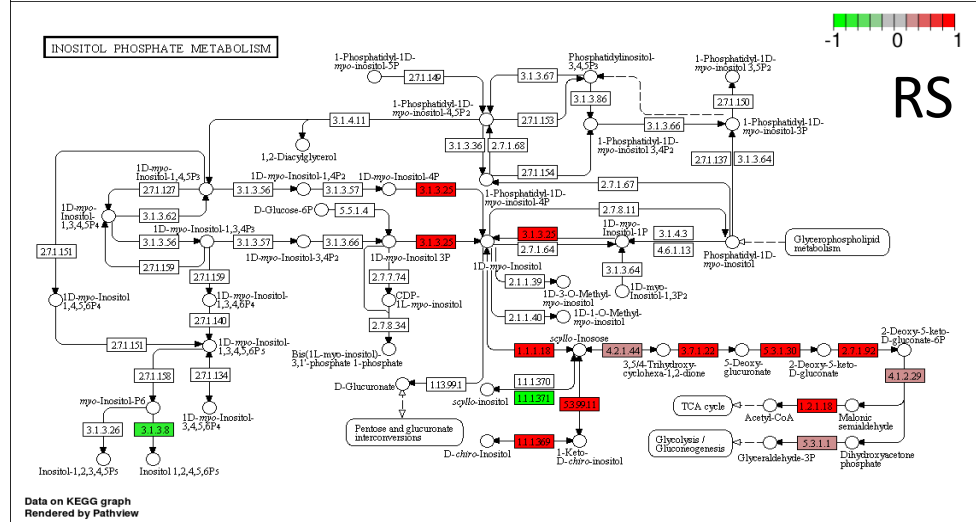

D

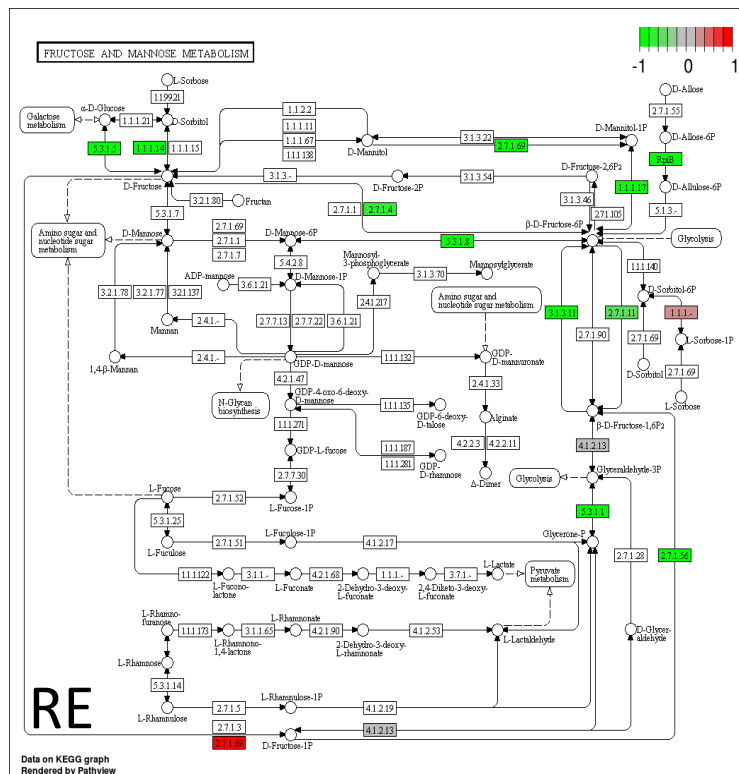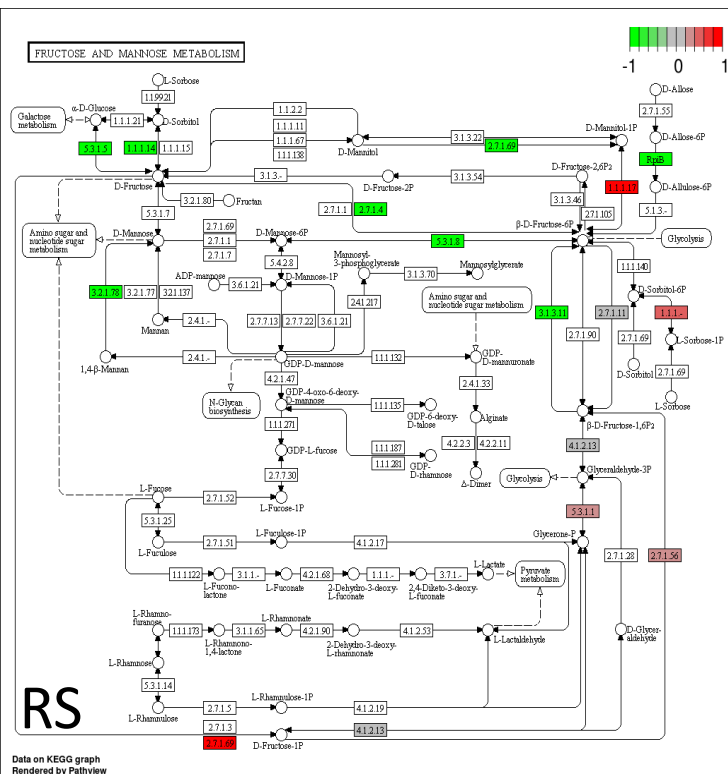

Supplement: S11 Fig — The regulated genes were mapped in the KEGG pathway and the diagram was accordingly adapted. The products encoded by up-regulated genes at middle stationary phase are highlighted in red while those by down-regulated genes are highlighted in green. RE: B. amyloliquefaciens FZB42 was grown in 1CM medium supplemented with the maize root exudates (RE); RS: B. amyloliquefaciens FZB42 was grown in 1CM medium supplemented with the maize root exudates (RE) and soil extract (SE). (PDF) [file pone.0142002.s011.pdf]
